# Active Visual Perception: Opportunities and Challenges

**DOI:** 10.3390/s26206544

**Published:** 2026-10-15

**Authors:** Yian Li, Xiaoyu Guo, Hao Zhang, Jiayu Chen, Shuiwang Li, Xiaowei Dai

**Affiliations:** 1 College of Computer Science and Engineering, Guilin University of Technology, Guilin 541006, China; liyian@glut.edu.cn (Y.L.); guoxiaoyu@glut.edu.cn (X.G.); zhanghao@glut.edu.cn (H.Z.); chennjiayu@163.com (J.C.); 2 College of Computer and Data Science, Putian University, Putian 351100, China

**Keywords:** active visual perception, active vision, next best view, perception and action loop, embodied intelligence, collaborative perception

## Abstract

Active visual perception enables an intelligent system to improve perception by controlling how visual evidence is acquired. However, differences in how studies define active perception and evaluate its benefits make approaches difficult to compare across tasks. This review first clarifies the field’s functional scope by examining whether an action is selected for its expected effect on subsequent visual evidence and whether the resulting observation informs a later decision. This definition distinguishes active observation acquisition from processing fixed inputs and guides the selection of representative literature. Within this scope, this review develops an analytical framework organized around four questions. It begins with what evidence the task requires, examines which actions can obtain that evidence and how a candidate observation should be valued, and considers how the next sensing action should be selected and executed. New observations then update the perceptual state and guide subsequent decisions, forming a closed loop that provides a common basis for comparing methods across applications. To examine how their benefits can be demonstrated, this review assesses whether existing datasets support observation selection, how interactive environments enable controlled comparisons, and what physical platforms reveal about execution feasibility. It further examines how baseline comparisons can establish whether adapting observations is necessary and whether informed selection offers benefits beyond simpler sensing strategies. These considerations underpin an evaluation framework that links improvements in visual evidence to downstream task outcomes while accounting for acquisition costs and risks. Its core value is to help isolate the contribution of sensing decisions and determine whether their benefits justify the resources required. The analysis concludes that predicted observation value does not guarantee that useful evidence can be acquired through feasible actions, while task success alone cannot establish the benefit of active observation selection. Further progress requires reliable value estimation and planning over longer decision sequences under physical and resource constraints. It also requires grounding semantic reasoning in acquired evidence and maintaining effective collaboration under imperfect communication, with these capabilities verified through reproducible evaluation of the complete perception and action loop.

## 1. Introduction

Visual perception is commonly treated as a process that begins after images or videos have already been acquired [1]. Under this assumption, the main challenge is to extract reliable representations, estimates, or predictions from a given input. This assumption is reasonable when the available observation contains sufficient evidence for the task, but it becomes restrictive when the evidence is incomplete, ambiguous, or poorly aligned with the current objective. A manipulation system cannot reliably estimate a grasp region that remains fully occluded [2,3]. An autonomous vehicle cannot directly assess a road user that remains outside its observable field because of occlusion or limited sensing range [4]. A tracking system cannot preserve target identity after visibility has been lost for a prolonged period, and a reconstruction system cannot recover a surface that has not been observed from a useful direction [5,6,7]. A stronger perception model may improve the interpretation of available data, but it cannot fully compensate for evidence that was never acquired. The system must therefore decide not only how to interpret its current observation, but also how to obtain a more useful one.

Active visual perception introduces this decision into the perception process. An active system controls some part of observation acquisition according to the current perceptual state or task requirement. The controlled variable may be a camera pose, gaze direction, platform trajectory, optical setting, sensing modality, physical interaction, or allocation of sensing responsibility among several agents [1,8]. The selected action changes the evidence available to the system, and the resulting observation influences later estimation, planning, communication, or task execution. Perception guides the sensing action, while the acquired observation updates the state on which later actions depend. Bajcsy described active perception through the purposeful selection and control of data acquisition strategies [9]. Aloimonos et al. further emphasized that visual perception can be improved by actively controlling the sensing process and the geometry of observation [10]. Ballard similarly emphasized controllable gaze and camera coordinates as essential components of visually guided behavior [11]. Subsequent work established that selective observation can reduce ambiguity and avoid unnecessary processing when different measurements do not provide equal value to the current task [1,12].

This principle now appears across a wide range of intelligent systems. Active reconstruction selects viewpoints that expose unobserved surfaces and improve geometric completeness [7,13,14]. Active localization, mapping, and exploration use camera or platform motion to reduce localization and mapping uncertainty, improve spatial coverage, and acquire more informative observations [15,16,17]. Visual search and active recognition acquire additional observations when the location, identity, or relevance of a target remains uncertain [18,19,20]. Active tracking redirects a camera, transfers responsibility between cameras, or changes platform motion to preserve visibility under target motion and occlusion [5,6,21]. Robotic manipulation may move the camera, reposition the robot, or interact physically with an object when the current view does not reveal the grasp region, contact state, or task progress [22,23,24]. Collaborative systems may coordinate which agent acquires complementary evidence or selectively communicate perceptually critical information when the local observation is insufficient [25,26].

Although these systems pursue different task outcomes, they repeatedly face the same technical dependency. A sensing action can be useful only when the system has identified what the current observation fails to provide. The system must then determine which controllable action can expose the required evidence, predict whether the resulting observation will improve perception or task execution, and decide whether the expected improvement justifies the associated cost. These decisions are often described through different task-specific terms. Reconstruction emphasizes coverage and completeness, localization emphasizes uncertainty and observability, search emphasizes target discovery, tracking emphasizes visibility preservation, manipulation emphasizes task success, and collaborative perception emphasizes complementary evidence and communication efficiency [6,7,16,19,23,26]. Similar mathematical quantities can therefore represent different forms of observation value. Information-based objectives, for example, can refer to uncertainty reduction in mapping or recognition, while task-oriented observation value may instead concern grasp verification or the usefulness of communicated evidence [18,26,27].

Existing reviews have established important foundations in active perception, sensor planning, robotic active vision, view planning, and active SLAM [1,8,28,29,30,31,32,33]. Despite their different scopes, these reviews share several limitations from the perspective of cross-task active visual perception. First, most of them organize the literature around a specific application domain or sensing problem, such as robotic active vision, view planning, or active SLAM. Consequently, methods that address similar observation–acquisition problems through different sensing actions are rarely compared within a common framework. Second, existing taxonomies mainly emphasize conceptual principles, sensor configurations, viewpoint-planning algorithms, or task-specific uncertainty criteria, but do not consistently connect the perceptual evidence missing from the current state, the action used to acquire that evidence, the expected value of the resulting observation, and the subsequent state update as a unified closed loop. Third, existing evaluations generally focus on final task performance or planning efficiency, with limited distinction among improvements in perceptual evidence, gains in downstream task performance, and the feasibility and resource costs of acquiring new observations. These shared limitations make it difficult to determine whether a reported improvement originates from a better observation–acquisition decision or from other components of the perception and decision-making pipeline.

To address these gaps, this review takes the observation–acquisition process, rather than a particular application domain or algorithm family, as the unit of cross-task comparison. Representative methods are examined according to four linked elements, including the perceptual evidence missing from the current state, the controllable action used to change the observation, the expected value of the resulting evidence, and the mechanism used to select, execute, and incorporate the action into the next system state. This structure is further connected to evaluation by distinguishing improvements in the acquired evidence from improvements in downstream task performance and from the feasibility and resource costs required to obtain those improvements. Table 1 summarizes how this perspective complements representative previous reviews.

The practical consequence is that reported performance gains can be difficult to attribute to the sensing decision itself. For example, a predicted camera pose may appear informative while remaining physically unreachable for the platform [30,34]. Likewise, improved final task performance does not necessarily demonstrate that a learning policy has acquired more useful visual evidence. In collaborative perception, higher detection accuracy may result from receiving additional information without showing that the message selection strategy itself is more effective than uniform communication [26,35,36]. These examples illustrate several possible failure points in the observation acquisition process. A system may misidentify the required evidence, select an unsuitable sensing action, misestimate the value of a candidate observation, or fail to execute the selected action under physical and resource constraints. A review centered on these dependencies is therefore needed to compare methods across tasks and to determine whether their reported benefits arise from effective active observation selection.

**Figure 1:**
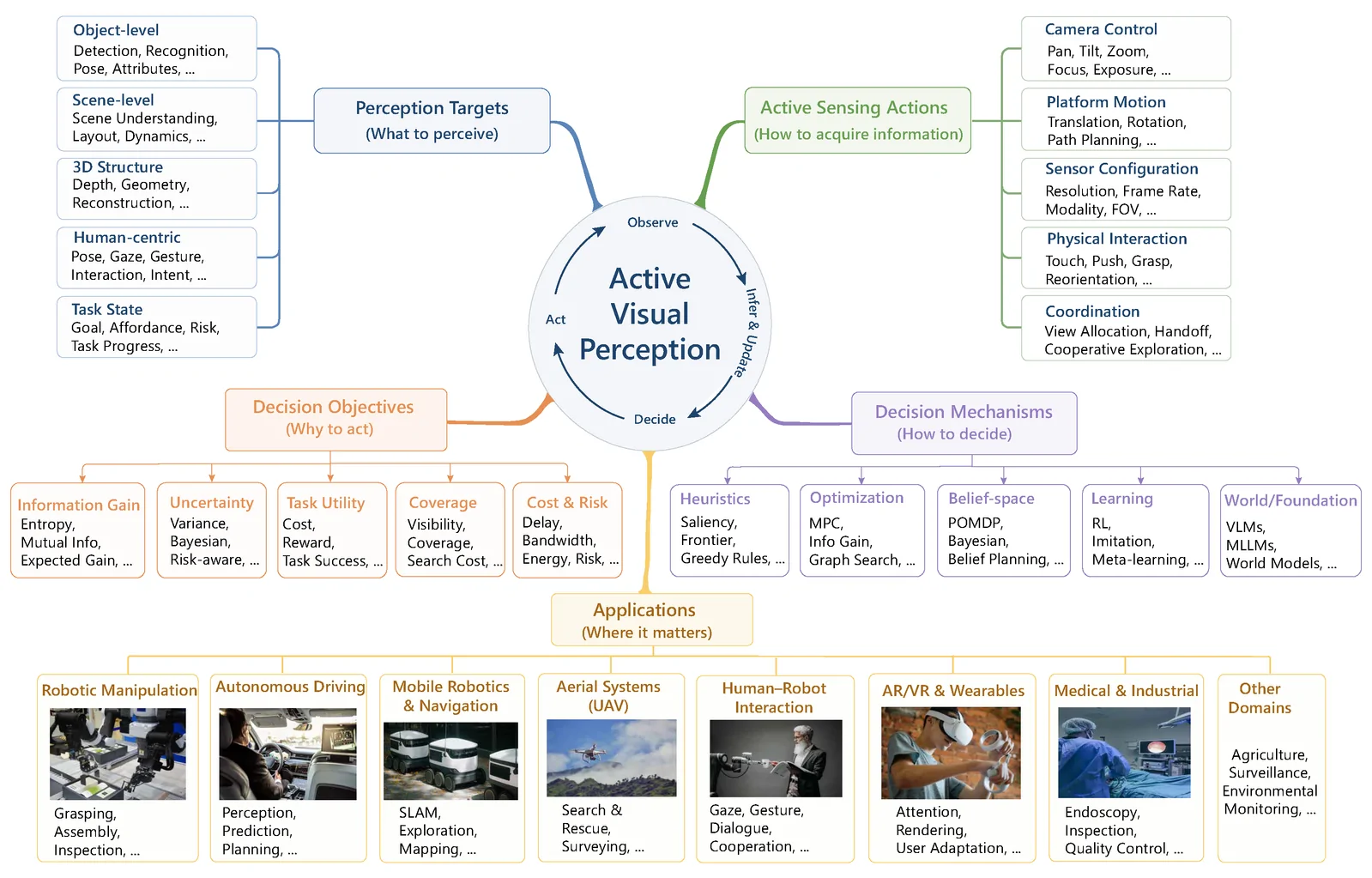
Conceptual framework of active visual perception linking perception targets, sensing actions, decision objectives, decision mechanisms, and application domains.

Such distinctions become even more important as active perception is increasingly integrated with vision language and vision language action models. These models can connect instructions with objects, regions, spatial relations, and task states, and they can use semantic reasoning to guide navigation, manipulation, or camera motion toward potentially relevant evidence [3,20,37,38,39]. Their semantic capability does not remove the classical active perception problem. A semantically plausible viewpoint may still be physically unreachable, unsafe, visually uninformative, or too expensive to obtain. A model may also produce a confident interpretation that is not adequately supported by the current observation. Active perception with foundation models must therefore connect semantic relevance with observability, uncertainty, feasibility, and evidence verification. Figure 1 provides an overview of the main perception targets, sensing actions, decision objectives, decision mechanisms, and application domains considered in active visual perception.

**Figure 2:**
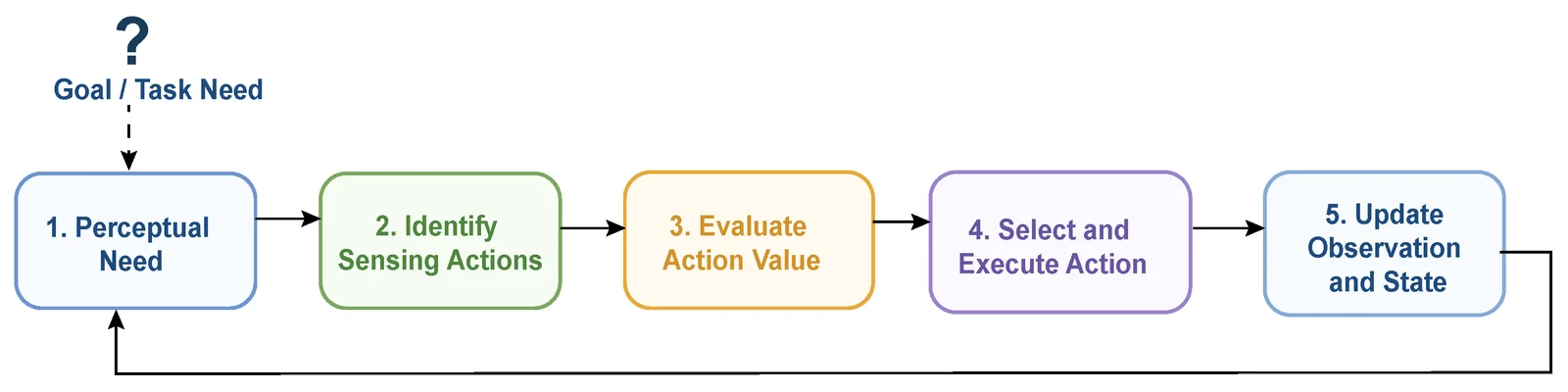
Closed-loop process linking perceptual needs, sensing actions, action evaluation, execution, and state updates.

This review examines active visual perception through four connected questions. First, the system must determine what visual evidence is required by the current task. Second, it must determine which sensing or embodied actions can change the observation and expose that evidence. Third, it must estimate the value of the candidate observation by considering its expected effect on perception and task execution together with its acquisition cost. Fourth, it must select and execute the next sensing action. After the new observation becomes available, the system updates its perceptual and task state, which closes the loop and determines the remaining perceptual need. These four questions describe successive decision dependencies, while the state update provides the feedback required for continued sensing. They do not assume that methods belong to mutually exclusive algorithmic categories. Geometric reasoning, probabilistic estimation, information-based planning, reinforcement learning, foundation models, and distributed coordination can each contribute to one or more stages of the loop. Figure 2 summarizes these dependencies. The selected sensing action changes the next observation, while the acquired evidence updates both the perceptual state and the remaining task requirement.

Based on the above definition and literature coverage, the main contributions of this review are summarized as follows:

- It clarifies the functional boundary of active visual perception by distinguishing it from passive visual processing, predetermined movement, fixed multimodal fusion, and general active learning. The defining criterion is whether an action is selected partly for its expected effect on subsequently acquired visual evidence.
- It presents active visual perception as a closed-loop process involving four connected questions. These questions concern what visual evidence is required by the task, which actions can change the current observation, how the value of a candidate observation should be estimated, and how the next sensing action should be selected and executed. This perspective supports comparison across reconstruction, localization, search, tracking, manipulation, sensor selection, and collaborative perception.
- It examines representative datasets, interactive environments, physical platforms, baselines, and evaluation criteria. The resulting evaluation perspective distinguishes improvement in the acquired visual evidence from improvement in the downstream task and from the motion, computation, communication, energy, time, and safety costs required to obtain that improvement.
- It identifies recurring challenges and research opportunities across active visual perception systems. These issues include task-guided evidence acquisition, uncertainty calibration, long-horizon decision-making, semantic verification, physical execution, collaborative sensing under imperfect communication, safety, and reproducible closed-loop evaluation.

The remainder of this review is organized as follows: Section 2 defines active visual perception, specifies its functional boundary, and explains the study selection criteria. Section 3 examines the perceptual evidence required by the task, the actions that change visual observation, the value of the acquired observation, and the selection of the next sensing action. Section 4 discusses datasets, interactive environments, physical platforms, baselines, metrics, and closed-loop evaluation requirements. Section 5 examines how the four parts of the sensing process appear in representative application settings. Section 6 analyzes recurring limitations across existing systems. Section 7 develops research directions from these limitations, and Section 8 concludes this review.

## 2. Definitions and Scope

### 2.1. Definition of Active Visual Perception

Not every change in visual input constitutes active visual perception. A mobile robot, for example, continuously receives new images while following a route, but these images may simply be a by-product of movement. The sensing process becomes active only when the robot chooses its action partly because of the visual evidence that the action is expected to provide. This distinction follows the early view of active perception as the purposeful control of sensing. Bajcsy emphasized that perception should include the selection and control of data acquisition strategies, while Ballard-related visual perception to controllable gaze and visually guided behavior [9,11]. Later work further described an active agent as a system that determines what information should be acquired, as well as where, when, and how it should be obtained [1].

Following this principle, this review defines active visual perception as a task-guided process in which a system selects an action partly for its expected effect on subsequent visual evidence. The system may be a robot, a vehicle, a camera network, or a group of sensing agents. What matters is that visual information is relevant to the current task, that the action responds to the current perceptual or task state, and that the resulting observation influences a later estimate, plan, or action. A predetermined camera trajectory therefore does not become active merely because it produces a sequence of different views. The same trajectory becomes relevant to active visual perception when it is chosen to reveal an occluded region, improve localization, preserve target visibility, or acquire evidence required for task completion. The defining property lies in the reason for selecting the action and in the role of the observation obtained after that action.

Within this visually grounded formulation, active perception is not restricted to a single imaging modality. Visual evidence may come from RGB, depth, thermal, event, or other imaging sensors, and other measurements may support the visual decision. Non visual modalities are included in this review only when they complement, guide, or are jointly selected with visual observations for a perceptual or task decision in which visual information remains an essential component. Systems that perform active sensing entirely through non visual measurements without affecting visual evidence acquisition are outside the present scope. A system may combine several modalities because different sensors remain reliable under different environmental conditions. However, using several sensors does not by itself make perception active. The process becomes active when the system decides which sensing modality should complement the visual observation, which region should be sampled, which viewpoint should be acquired, or which information should be transmitted. A system that always combines the same predetermined measurements performs multimodal fusion, whereas a system that changes its sensing resources according to the current information need performs active observation selection. The same distinction applies to recent vision language systems. Language and semantic reasoning become relevant when they guide the acquisition of additional evidence, such as deciding where the system should look, move, revisit, or interact. A model that reasons only over a fixed image changes the interpretation of existing evidence, but it does not change how new evidence is obtained.

### 2.2. Included Forms of Observation Control and Tasks

The definition above leads to several ways in which a system can improve its observations. The most direct approach is to change the geometric relationship between the sensor and the scene. Camera translation, rotation, gaze control, viewpoint selection, zoom, and viewing distance determine which regions are visible, how large an object appears, and which geometric constraints can be recovered from the image. This form of control is central when the missing evidence can be obtained from another direction or scale. When changing the viewpoint alone is insufficient, the system may instead change the sensing configuration. Exposure, focus, resolution, frame rate, field of view, sampling density, and modality selection affect the quality, frequency, and type of evidence that can be acquired. These decisions are especially important when observation quality varies with illumination, motion, distance, or sensor limitations.

Some information cannot be obtained by changing the camera settings alone because the sensor must move through the environment or the scene itself must be changed. Mobile robots may therefore select trajectories that improve feature visibility, reduce localization uncertainty, extend spatial coverage, or maintain a target within view. Manipulation systems may go further by pushing, rotating, grasping, or repositioning an object so that hidden surfaces or task-relevant regions become observable [22,23,24]. In distributed systems, the missing evidence may already be visible to another sensor or agent. The decision then concerns which agent should observe a region, which camera should continue tracking a target, which information should be transmitted, and when sensing responsibility should be transferred [6,25,26]. Camera control, platform motion, physical interaction, and collaborative sensing therefore represent different ways of addressing the same underlying problem, which is how to change the available evidence when the current observation is insufficient.

These forms of observation control recur across several research tasks. In active reconstruction, the system selects new views because the current model contains unobserved or poorly reconstructed surfaces [7,13,14]. In active localization and mapping, movement is selected not only to reach a destination, but also to obtain features that improve pose estimation, map quality, spatial coverage, or loop closure [15,16,17]. Visual search and active recognition acquire additional views because the location, identity, or relevance of a target remains uncertain [18,19,20]. Active tracking faces a different source of uncertainty because the target and the observer may both move. Camera orientation, platform motion, or camera assignment must therefore be adjusted to preserve visibility and avoid target loss [5,6,21]. Robotic manipulation uses observation control when successful execution depends on evidence about object pose, grasp regions, contact, free space, or task progress. Collaborative perception extends the same reasoning to multiple agents by deciding how observation, processing, and communication responsibilities should be divided. Although these tasks pursue different outcomes, they are included for the same reason. In each case, the system selects an action because of the evidence that the action is expected to make available.

### 2.3. Boundary with Neighboring Research Areas

The same criterion also clarifies the boundary between active visual perception and several neighboring fields. Conventional image and video analysis begins with an observation that has already been acquired. Detection, segmentation, recognition, and visual question answering may improve how that observation is interpreted, but they remain outside the present scope when they do not influence later data acquisition. Attention mechanisms do not change this distinction. Selecting image features or regions inside a fixed input changes internal processing, whereas active visual perception changes the evidence obtained from the environment. Offline multi view processing is excluded for the same reason when all views are captured according to a predetermined procedure. The availability of several images may improve recognition or reconstruction, but active selection is absent unless the system determines which additional view should be acquired.

**Figure 3:**
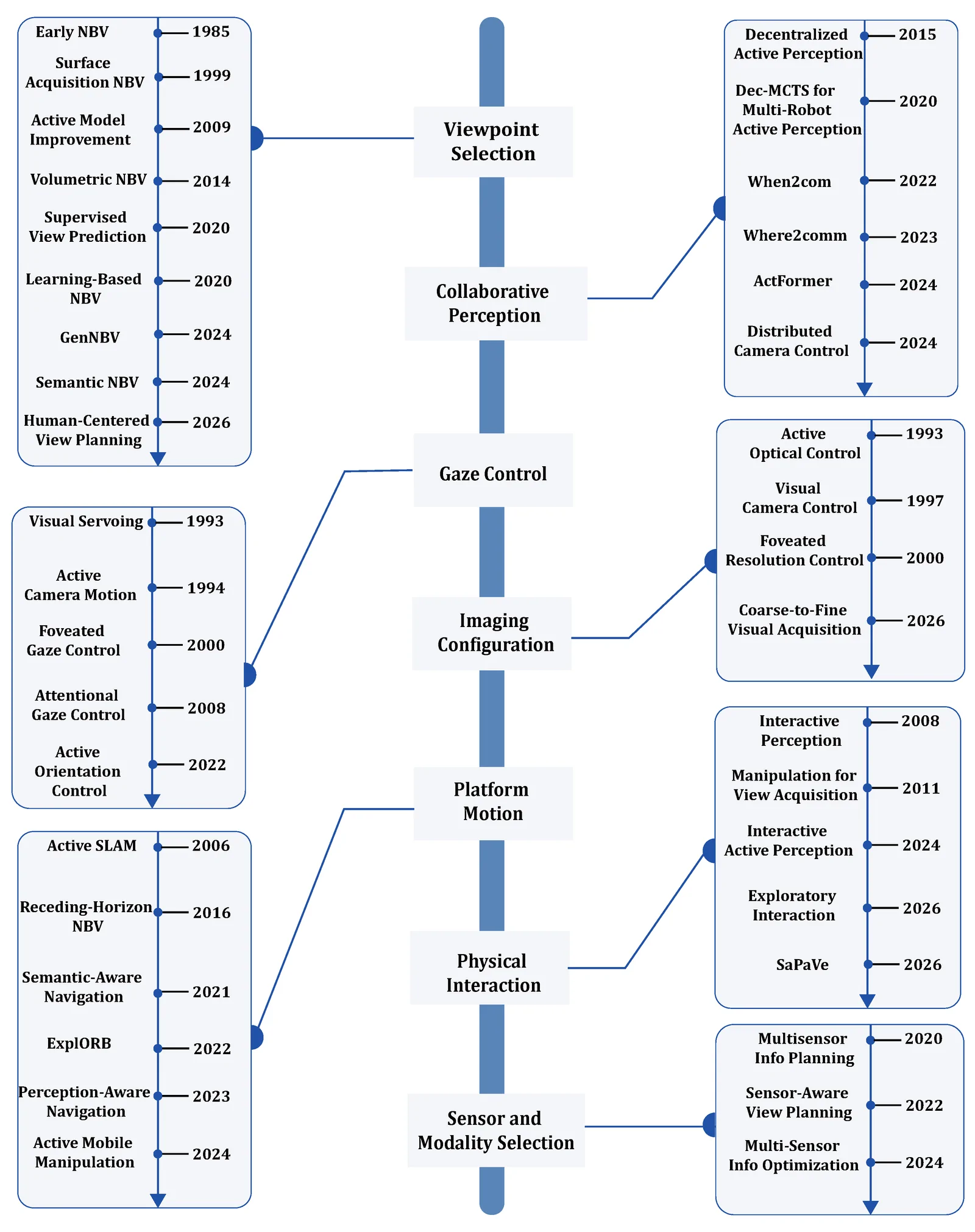
Representative milestones in active visual perception organized by observation control mechanism.

Active learning also involves the selection of information, but it operates at a different stage. It usually chooses existing samples for annotation or model training, while active visual perception chooses sensing or embodied actions that alter future observations. Other neighboring fields overlap more directly with the present scope. Active sensing includes decisions about modalities beyond vision and is therefore broader than active visual perception. Embodied perception studies how physical movement and interaction shape perception, although it does not always require the agent to choose an action for the purpose of obtaining information [40]. Perception aware planning incorporates visibility, observability, or feature quality into motion planning and becomes part of active visual perception when these considerations directly affect the acquisition of visual evidence. Multimodal fusion becomes active only when the system selects the sensor, observation, sampling pattern, or transmitted information rather than combining a fixed set of inputs. These boundaries are not determined by terminology alone. A study is included when its selected action changes the acquisition or allocation of visual evidence and when the resulting evidence contributes to a later perceptual or task decision. Figure 3 highlights representative developments in observation control, from camera and gaze control to platform motion, physical interaction, adaptive sensing, and collaborative perception.

### 2.4. Literature Coverage and Study Selection

The evidence base was developed through iterative searches in Google Scholar and Web of Science and was last updated on 5 September 2026. The search strategy was designed to identify representative studies that provide broad coverage of the main forms of observation control and decision-making in active visual perception, rather than to support an exhaustive systematic review. The search began with the broad terms “active vision,” “active visual perception,” and “active sensing.” These terms were then combined with task-specific and action-specific expressions, including “next best view,” “active 3D reconstruction,” “active SLAM,” “active exploration,” “active visual search,” “active object recognition,” “active camera control,” “gaze control,” “active tracking,” “interactive perception,” “active perception for robotic manipulation,” “collaborative active perception,” “multi-agent active perception,” and “active sensor selection.” Backward and forward citation tracing from foundational reviews and representative recent studies was used to identify influential earlier work, alternative terminology, and studies addressing multiple task settings or forms of observation control.

A study was included when three conditions were satisfied. First, visual evidence was required for the current perceptual estimate or task decision. Second, the system selected a sensing or embodied action partly because of its expected effect on subsequent visual evidence. Third, the newly acquired observation contributed to a later estimate, plan, communication decision, or task action. The selected action could control the viewpoint, gaze direction, camera configuration, platform motion, temporal or spatial sampling, sensing modality, physical interaction, sensor assignment, or communication of visual evidence.

Studies were excluded when they only processed fixed images or prerecorded videos, followed a predetermined trajectory without adapting to the perceptual state, combined a fixed set of sensor inputs, or selected existing samples for annotation or model training. General motion planning was also excluded when visibility, observability, or visual information quality did not influence the selected action. Offline multiview reconstruction was retained only when the system determined which additional observation should be acquired. These criteria apply the functional definition in Section 2 consistently across reconstruction, localization, search, tracking, manipulation, and collaborative perception. The main literature areas and representative search terms are summarized in Table 2.

## 3. A Taxonomy from Perceptual Need to Sensing Action

Active visual perception begins when the current observation cannot provide sufficient evidence for the next perceptual or task decision. The missing evidence may concern an object hidden by occlusion, a surface that has not been reconstructed, an uncertain camera pose, a target that is about to leave the field of view, or a task region that cannot be inspected from the current position. In distributed systems, the required evidence may already be available to another sensor or agent but remain inaccessible because it has not been requested or transmitted. The system must therefore determine what evidence is needed, how the observation can be changed, whether the expected improvement is worth the associated cost, and which sensing action should be executed.

These questions describe successive stages of the active visual perception loop. The task first determines which part of the current perceptual state remains incomplete or unreliable. The system then identifies the sensing actions that may expose the required evidence. Candidate observations are evaluated according to their expected contribution to perception and task execution, together with the motion, computation, communication, energy, and safety costs required to acquire them. A decision mechanism finally selects an action and updates the state after the new observation becomes available. The effectiveness of an active perception method depends on all of these stages because an informative objective is of little use when the required action is unreachable, while a powerful decision model cannot compensate for a state representation that fails to describe the information needed by the task.

### 3.1. Perceptual Evidence Required by the Task

The first question in active visual perception concerns the evidence that is missing from the current observation. This question must be answered before a useful sensing action can be selected because different forms of perceptual insufficiency require different responses. A target that is hidden behind an obstacle may require a change in viewpoint, while a distant target may require a narrower field of view or higher spatial resolution. An uncertain object identity may require an observation that reveals a discriminative surface, whereas an uncertain robot pose may require visible landmarks or a loop closure. The required evidence is therefore determined jointly by the current perceptual state and the task that the system is attempting to complete.

#### 3.1.1. Occluded and Unobserved Content

Occlusion and incomplete scene coverage are among the most direct reasons for acquiring a new observation. In active reconstruction, an incomplete model indicates that some surfaces have not been observed or have been reconstructed with insufficient geometric support. The system therefore searches for a viewpoint that exposes these regions and provides useful depth or appearance measurements [7,13,14,41,42,43]. The same problem appears in robotic manipulation when an object, grasp region, or contact area is hidden by clutter. Changing the camera position may reveal the required region, but some occlusions cannot be removed through camera motion alone because every reachable viewpoint remains blocked.

Visibility also changes over time. In active tracking, a target may approach the image boundary, pass behind another object, or become difficult to distinguish from the background. The system must anticipate this loss of evidence and redirect the camera or platform before the target disappears [5,6,21]. Visual search faces a related problem because the target may lie outside the current field of view or inside a region that has not yet been inspected [18,19,44]. Although these tasks have different outputs, they share the need to expose visual content that is currently unavailable.

#### 3.1.2. Uncertain State and Task Progress

The current observation may contain the relevant object or region but still fail to support a reliable decision. Active recognition methods acquire additional views when several object hypotheses remain consistent with the current image [18,45]. Active localization and mapping methods seek observations that reduce uncertainty in the robot pose, map structure, or data association [15,16,31,32]. In these settings, the missing evidence is not simply an invisible region. It is the information needed to distinguish between competing estimates of the current state.

Task execution introduces further information requirements. A manipulation system may need evidence about object pose, grasp stability, contact, free space, or task progress rather than a complete description of the scene. A navigation system may need to identify a traversable route or confirm a semantic landmark. Recent vision language systems extend these requirements to open vocabulary objects, spatial relations, and instruction dependent regions [20,37,38]. Their sensing decisions must still determine whether the current observation supports the requested conclusion or whether additional visual evidence is necessary. Semantic plausibility alone cannot replace observation because a model may produce a confident interpretation without having viewed the relevant region.

#### 3.1.3. Complementary Evidence from Sensors and Agents

Within visually grounded active perception, another sensing modality may provide complementary evidence when the current visual observation is insufficient. A visible light camera can provide texture and semantic detail, while depth sensors and LiDAR can provide geometric structure. Thermal sensing can reveal people and heat patterns under poor illumination, and radar can remain useful when visual conditions deteriorate. The need for an additional modality arises when the current sensor cannot reliably measure the property required by the task. Reflective surfaces, darkness, adverse weather, long distance, and rapid motion can all change which sensing modality provides the most useful complementary evidence for the visually grounded task.

A similar situation occurs in collaborative perception. Another vehicle, robot, or camera may already have an unobstructed view of a region that is hidden from the current agent. The perceptual need can then be addressed through observation assignment or communication rather than additional movement. Collaborative driving systems select spatial regions, features, or queries that complement the local observation [26,35,36]. Multi-camera systems can transfer target tracking between cameras, while multi-robot systems can divide unexplored areas or direct different agents toward complementary viewpoints [6,25,46]. The useful evidence is determined by what the current system lacks, not by the total amount of information available across the network.

### 3.2. Actions That Change Visual Observation

Once the required evidence has been identified, the system must determine how the observation can be changed. Some information can be obtained by redirecting the camera, while other information requires platform motion, a change in imaging configuration, physical interaction, or assistance from another agent. These actions differ in their physical effect, execution cost, and degree of risk. They also determine which future observations are reachable and therefore constrain the decisions that can be made later in the closed loop.

#### 3.2.1. Viewpoint, Gaze, and Platform Motion

Changing the geometric relationship between the sensor and the scene is the most common way to acquire new evidence. The system may change the camera position, orientation, viewing distance, or platform pose. Classical next best view methods evaluate candidate camera poses according to visibility, surface coverage, or uncertainty [7,41,47,48]. Their decisions are interpretable because each candidate corresponds to a concrete geometric configuration. However, the result depends strongly on how candidate poses are generated. A sparse candidate set can exclude useful views, while a dense set increases the cost of visibility prediction, collision checking, and motion planning.

Learning-based methods predict a viewpoint or its expected value from the accumulated observation state [13,14,49]. They can represent complex relations between partial geometry and future coverage without evaluating every possible view online. They can also move beyond manually designed camera orbits. One hybrid pipeline first acquires a single next-best view and then predicts a set of subsequent views with a multiview-conditioned network trained through long-tail sampling [50]. Their predictions nevertheless depend on the geometry, sensor characteristics, and action constraints represented during training. A predicted viewpoint may appear informative in image space but remain unreachable or unsafe for the physical platform.

The value of a viewpoint also changes with the task. Human centered view planning seeks observations that preserve useful views of a person rather than maximizing generic scene coverage [51]. Sensor aware inspection considers whether a surface will produce a valid measurement rather than only whether it falls inside the field of view [52]. Plant inspection and semantic reconstruction prioritize structures or object classes that matter to the task [53,54]. These studies show that geometric visibility is only the starting point. A useful viewpoint must reveal the right evidence at a quality that supports the intended decision.

Visual servoing provides a classical feedback mechanism for coupling visual measurements with camera or robot motion [55]. Gaze control changes the direction of observation without necessarily changing the camera position [56]. A pan and tilt camera can follow a moving target while the platform remains stationary, and a mobile robot can rotate its camera or body toward useful landmarks without changing its translational route. Camera motion can preserve target visibility, but it also produces background motion and control error that must be separated from target motion [5,57]. Foveated systems add another decision because high resolution sensing is concentrated near the fixation point, while the periphery is observed at lower resolution [21]. Active localization methods also use orientation control to observe stable or informative features even when the route remains unchanged [17,58].

Platform motion extends viewpoint control because the same movement changes both the visual observation and the physical state of the task [59]. Active localization and mapping methods move toward unexplored regions, informative landmarks, or potential loop closures [15,16]. Perception aware navigation favors routes that maintain stable visual features or semantically useful regions [60,61]. Mobile manipulation must further consider whether a better observation justifies additional travel, collision risk, and interruption of the main task [34]. A camera pose that cannot be reached through a safe and efficient trajectory has limited practical value even when its predicted visual quality is high.

#### 3.2.2. Imaging Configuration and Adaptive Sampling

Visual evidence can also be changed without moving the camera. Focus, zoom, aperture, exposure, field of view, spatial resolution, frame rate, and temporal sampling determine the quality and frequency of the measurements delivered to the perception system. Early active vision research treated these controls as part of the sensing process because a different viewpoint cannot correct blur, saturation, poor scale, or limited depth of field [12,62]. Recent manipulation systems have renewed interest in coarse to fine sensing, where a broad observation identifies a relevant region and a focused observation supports precise action [3].

Optical and temporal controls are closely coupled to perception failures. Zoom can provide more detail but narrows the field of view and increases the risk of target loss. A longer exposure can improve low light appearance but also increase motion blur. A higher frame rate can improve tracking of rapid motion but consumes additional computation, bandwidth, and energy. The same sensing configuration is therefore not optimal throughout a task. A system may begin with wide and inexpensive observations, allocate greater resolution when uncertainty becomes concentrated in a small region, and reduce the sampling rate when the scene changes slowly.

These controls remain less developed than viewpoint planning. Many systems mention adjustable resolution or zoom but treat them as fixed hardware settings rather than actions selected from the current perceptual state. Most visual benchmarks also assume a fixed frame rate and fixed image quality. This assumption hides an important capability of physical systems because changing the sensing configuration may sometimes provide more useful evidence at a lower cost than moving the entire platform.

#### 3.2.3. Physical Interaction

Camera and platform motion cannot reveal all task-relevant information. In cluttered environments, every collision free viewpoint may remain occluded, and some object properties only become observable after the object has been moved or touched. Interactive perception addresses this limitation by changing the observed object or the surrounding environment. A robot may push an occluder, rotate an object, open a container, grasp and reorient a part, or establish contact that reveals material or mechanical properties [22,23,24,63].

Interaction can expose evidence unavailable through visual observation alone. Repositioning an object can reveal a hidden surface and improve pose estimation. Interaction-driven reconstruction extends this idea to object interiors by manipulating articulated parts to expose geometry that cannot be scanned from external viewpoints alone [64]. Repeated contact can reveal articulation, deformability, mass distribution, or tool function. Predictive visuo-tactile perception selects informative pushing and pulling actions to infer indirectly observable physical properties, including mass, center of mass, and friction [65]. Recent systems coordinate exploratory actions with task directed behavior so that the robot first reduces uncertainty and then performs the intended manipulation [24,66]. The sensing action and the task action may therefore become part of the same process rather than two separate stages.

The additional information comes with greater physical risk. An interaction may damage an object, move it into a less observable configuration, create an irreversible state, or interfere with later task execution. Contact outcomes are also difficult to predict accurately. Interactive perception is therefore most appropriate when the required evidence cannot be obtained safely through camera or platform motion and when the expected information justifies the possibility of disturbing the scene.

#### 3.2.4. Collaborative Observation and Communication

Multiple agents can enlarge the observable region and reduce individual blind spots, but their observations become useful only when sensing responsibilities are coordinated. Multi-robot exploration assigns different regions, frontiers, or observation tasks to different agents and coordinates their motion to improve exploration efficiency and sensing coverage [67,68]. Multi-camera tracking determines which camera should observe a target and when another camera should take over. Collaborative driving allows vehicles and roadside units to provide evidence about regions that are occluded from the local vehicle.

The action in these systems may involve movement, sensor assignment, message selection, or communication timing. Classical decentralized methods seek informative joint behavior without relying entirely on a central controller [25,46]. Recent collaborative perception systems select spatial regions, features, or queries rather than transmitting all available data [26,35,36]. Distributed camera control coordinates viewpoints so that target visibility and identity can be maintained across a wider area [6].

The benefit of collaboration comes from complementary evidence rather than the number of sensors alone. Repeated transmission of information already available to the receiving agent wastes bandwidth and processing resources. Coordination becomes more difficult when agents have different sensing capabilities, uncertain relative poses, delayed messages, or inconsistent local maps. The system must therefore determine who should observe, what should be transmitted, whether the information will remain valid when it arrives, and how perception should continue when communication becomes unreliable.

### 3.3. Value of the Acquired Observation

Changing the observation is useful only when the newly acquired evidence improves perception or task execution. The value of an observation cannot be determined from visual change alone because a different image may contain no additional task-relevant information. Active perception systems therefore predict how a candidate observation will affect visibility, uncertainty, geometric quality, task outcome, or resource consumption. Different objectives emphasize different aspects of this improvement, and their suitability depends on what the system currently needs to know.

#### 3.3.1. Visibility, Coverage, and Geometric Quality

Visibility provides the most direct measure of whether a sensing action reveals the required target, surface, or region. A tracking system can seek to keep a target inside the field of view, a manipulation system can seek an unobstructed view of a grasp region, and a reconstruction system can seek viewpoints that expose unobserved surfaces. Coverage extends this reasoning from a single target to the amount of scene or object geometry observed by the system. Surface completeness, valid depth, map coverage, and frontier reduction are common measures in reconstruction and exploration.

These objectives are easy to interpret, but visibility alone does not guarantee useful evidence. A surface may be visible but too distant, blurred, reflective, or poorly illuminated for reliable measurement. A person may occupy a large image region but appear from an angle that does not support pose estimation or identification. A large unknown region may increase map coverage while contributing little to the current task. Sensor aware and target-oriented methods address these limitations by combining geometric visibility with measurement validity, semantic relevance, or task affordance [51,52,54,69].

Coverage can also conflict with localization quality. An exploration policy may repeatedly enter unknown areas even as pose uncertainty increases. Conversely, a localization-oriented policy may remain near familiar landmarks and fail to expand the map. Active localization and mapping methods therefore combine exploration value with feature quality, trajectory cost, and loop closure potential [16,61]. The central question is not how much new space can be observed, but whether the added coverage can be obtained while maintaining a reliable perceptual state.

#### 3.3.2. Information Gain and Uncertainty Reduction

Many active perception methods value an action according to the expected reduction in uncertainty. The uncertain state may describe object identity, surface occupancy, robot pose, map structure, target location, or semantic belief. The system predicts how a candidate observation will change this state and prefers observations expected to distinguish competing hypotheses or concentrate the posterior estimate.

This principle appears across different tasks. Active recognition selects views that separate object hypotheses [18]. Active reconstruction values observations according to uncertainty over occupancy, surface geometry, and radiance field representations, while hierarchical uncertainty can be used to guide next best viewpoint selection [70]. Active localization and mapping use pose covariance, map uncertainty, or information matrices to estimate the value of candidate movements [27,31,71]. Search systems use spatial or semantic beliefs to determine which region is most informative to inspect next. The mathematical form may be shared, but the meaning of uncertainty depends on the state representation used by the system.

The quality of an information driven decision depends on the calibration of that representation [72]. A system can be confidently wrong when the scene differs from the training data or when the observation model ignores sensor failure. A geometric model may overestimate the value of a reflective surface, while a vision language model may produce high semantic confidence without sufficient visual support. Inaccurate uncertainty can cause the system to seek the wrong evidence and reinforce an incorrect belief. Evaluation should therefore compare predicted observation value with the improvement that is actually obtained after the action, rather than reporting only the final task result.

#### 3.3.3. Downstream Task Utility

Task utility provides active perception with a task-specific evaluation criterion. Its core principle is that a sensing action should be selected according to whether the resulting observation can improve subsequent decisions, rather than merely increase visibility or reduce general uncertainty [73,74,75]. This principle takes different forms across tasks. In manipulation, a useful viewpoint provides evidence that improves grasping, insertion, contact verification, or gripper pose selection [76,77,78,79]. In navigation, active target perception uses real time detection feedback and task constraints to adjust the robot position and provide a suitable viewpoint for subsequent manipulation [80,81,82,83]. In visual search, next best view planning directs the sensor toward viewpoints that reveal occluded targets and improves search decisions under uncertain detections [19,84,85]. In collaborative perception, communication is useful when shared information fills missing perceptual evidence and consequently improves the receiving agent’s decisions [86,87,88,89]. These examples demonstrate that the value of an observation depends on how the evidence it provides influences task execution.

However, the final task outcome alone does not reveal whether an improvement results from better observations or from other components of the policy. A reinforcement learning policy may achieve a higher success rate because it has learned favorable motion patterns rather than because it acquires better visual evidence. A vision language action model may also benefit from stronger semantic or action priors even when active camera control contributes little. Controlled comparisons between active and fixed camera configurations can help isolate the contribution of viewpoint adaptation [2,90]. Evaluation should therefore report observation quality, sensing decisions, controller performance, and final task outcomes separately [91].

This distinction is especially important in recent manipulation systems that tightly couple visual acquisition with physical action [24,92]. The dual arm DAVMM model predicts eye and hand actions simultaneously for manipulation under occlusion [93]. SaPaVe jointly optimizes semantic camera control and manipulation through separate but coordinated action spaces [39], while ActiveVLA uses coarse to fine region localization, active view selection, and spatial refinement to support precise manipulation [3]. In these systems, the useful observation depends on the next physical action, while the action also depends on the evidence acquired from the selected viewpoint. Active perception therefore remains coupled to task execution throughout the closed loop.

#### 3.3.4. Operational Cost and Risk

An observation that improves perception may still be unsuitable when the cost of acquiring it is too high. Camera motion, robot travel, sensing, model inference, communication, and physical interaction all consume limited resources. These costs determine whether active perception improves the complete system rather than only the accuracy of an isolated perception model.

Motion cost can be represented by path length, rotation, action count, execution time, or interruption of the main task. Computational cost includes candidate generation, observation prediction, optimization, and network inference. Energy is critical for UAVs, mobile robots, and long-term monitoring systems. Communication cost affects collaborative perception because transmitting dense features or raw images can exceed available bandwidth. Risk includes collision, unstable viewpoints, object damage, privacy intrusion, and actions that leave the system without a safe recovery path.

Many methods combine expected benefit and cost within a weighted objective or constrained optimization. A single combined score can simplify action selection but makes comparison difficult because the meaning of each weight changes across platforms. One meter of motion has a different cost for a ground vehicle, a UAV, and a wrist mounted camera. Reporting task improvement and individual costs separately provides a clearer account of whether the method remains useful when the resource budget or deployment platform changes.

### 3.4. Selection of the Next Sensing Action

After the system has defined the available actions and the expected value of their resulting observations, it must determine which action should be executed. The difficulty of this decision depends on the number of candidate actions, the uncertainty of the current state, the availability of an observation model, and the time over which future consequences must be considered. Simple and well structured tasks can often be handled through explicit rules or geometry, while uncertain and long-term tasks require probabilistic reasoning, planning, learning, or combinations of several mechanisms.

#### 3.4.1. Geometric and Rule-Based Decisions

Rule-based methods select sensing actions through designed priorities, thresholds, and state transitions. A camera may recenter a target when it approaches the image boundary. A robot may rotate toward a landmark when the number of visible features falls below a threshold. A search system may inspect salient regions before less relevant regions. These decisions are computationally efficient and easy to interpret when the operating conditions are well defined.

Geometry-based methods use visibility, occlusion, projection, surface orientation, baseline, reachability, and collision constraints to evaluate candidate actions [94]. They remain effective in reconstruction and inspection because the relation between camera pose and surface observation has a clear physical basis [7,52]. They do not require large training datasets, and their failures can often be traced to a specific geometric assumption.

Their limitations become more apparent when the scene is dynamic, deformable, semantically ambiguous, or difficult to model. A geometric policy requires a sufficiently accurate partial map or object representation. It may also treat all visible surfaces as equally measurable even when sensor response depends on material, lighting, or motion. Practical systems often preserve geometric constraints for safety and feasibility while using other mechanisms to estimate task relevance or observation quality.

#### 3.4.2. Probabilistic and Information-Based Decisions

Probabilistic methods maintain a belief over uncertain states and predict how candidate observations will modify that belief. Bayesian active recognition selects views that distinguish object hypotheses [18]. Active localization and mapping propagate pose and map uncertainty through possible controls. Belief-based reasoning is well suited to partial observability because the system can represent several possible states instead of committing immediately to one estimate.

Building on such belief representations, information-based active perception compares sensing actions according to their expected reduction in task-relevant uncertainty. Active SLAM methods implement this principle using information gain or KL divergence to estimate how future observations may reduce pose and map uncertainty [27,95]. Recent work extends this formulation to dynamic semantic environments by combining mutual information with hierarchical NBV decisions to improve path planning, mapping efficiency, and tracking robustness [96]. Fisher information applies the same principle to active view selection in radiance field mapping [97]. Submodular formulations support coordinated observation selection under constraints involving multiple sensors or multiple robots [98,99]. These criteria provide a common framework for connecting sensing actions with expected uncertainty reduction, but their reliability depends on the belief and observation models from which their scores are computed. Poorly represented uncertainty or omitted sensor failure modes may therefore cause a mathematically optimal action to provide little useful perceptual evidence [72]. This dependence becomes more consequential as the belief expands to include several coupled sources of uncertainty.

A realistic belief may need to describe robot pose, map uncertainty, target identity, object motion, sensor reliability, and task state at the same time. Exact prediction becomes difficult as the state and action spaces grow. Practical methods therefore simplify the belief, approximate future observations, restrict candidate actions, or consider only a limited future horizon. These approximations reduce computation but must be reported clearly because they determine which uncertainty sources influence the final decision.

#### 3.4.3. Search and Receding Horizon Planning

A single sensing action may have consequences that only become visible after several later actions. A locally informative view can move the system into a poor position for subsequent observation, while an initially less informative action can create access to a sequence of valuable views. Search and planning methods address this dependence by evaluating candidate sequences, graph paths, frontiers, or trajectories [100,101,102,103].

Graph search- and sampling-based methods can compare discrete viewpoints and motion sequences. For multi-target active tracking, Monte Carlo tree search can be combined with target-motion prediction to select camera actions [104]. Trajectory optimization can handle continuous platform or camera motion. Model predictive control evaluates actions over a limited future horizon, executes the first action, and replans after the next observation [15,61,105,106,107]. This repeated update preserves responsiveness to newly acquired evidence and reduces the computational burden of full long-term planning.

Planning quality depends on both the candidate generator and the model used to predict future observations. A planner cannot select a viewpoint that is absent from the action space. It can also overestimate a candidate when the prediction ignores blur, calibration error, reflective surfaces, dynamic occlusion, or communication delay. The planning mechanism must therefore be evaluated together with the state representation and observation model on which its predictions depend.

#### 3.4.4. Learning-Based Policies

Supervised methods learn a mapping from the current observation state to a next viewpoint or an estimate of viewpoint utility [14,49]. By learning from labelled viewpoints or supervision derived from view quality, these methods can reduce the need for exhaustive online evaluation of candidate observations. Imitation learning and hybrid behavior cloning and reinforcement learning approaches can acquire active sensing strategies from demonstrations and interaction, including visual search, tracking, focusing, and task coupled gaze control [2,108,109]. Both paradigms enable efficient policy inference, but their performance depends on how well the training data represent the observation states, sensing actions, and task conditions encountered at deployment.

Reinforcement learning learns sensing policies through interaction and can account for rewards that appear only after several actions. It has been applied to reconstruction, active localization and mapping, semantic navigation, tracking, robotic manipulation, and camera networks [13,60,110,111,112,113]. A reward can combine perceptual improvement, task success, and action cost, allowing the policy to adapt its behavior to long-term consequences.

The main difficulty is transfer. A policy may exploit visual appearance, motion dynamics, action discretization, or reward structure specific to the training environment. Changes in camera calibration, actuator delay, lighting, object dynamics, or sensor noise can invalidate the learned relation between action and observation. Evaluation should therefore include unseen environments, perturbations, and real platforms rather than only new episodes generated from the same simulator [114].

#### 3.4.5. Semantic Reasoning with Foundation Models

Foundation models provide language conditioned representations and broad semantic knowledge that can help identify which objects, regions, or relations are relevant to a task. Open vocabulary navigation methods use uncertainty or semantic relevance to guide exploration [20]. ProFocus generates targeted visual queries when the current evidence is insufficient [37]. Embodied Reasoner connects observation, reasoning, and action over embodied trajectories [38]. ActiveVLA and SaPaVe further connect semantic reasoning with camera control and manipulation [3,39].

These systems extend active perception beyond fixed categories and predefined task states. However, semantic confidence is not equivalent to visual evidence. A model may generate a plausible conclusion even when the relevant object or region has not been observed. The sensing process must distinguish prior semantic knowledge from evidence acquired in the current environment and must verify that a proposed viewpoint is reachable, safe, and likely to resolve the uncertainty.

Latency and reproducibility also affect closed-loop use. A large model may delay sensing decisions, and external model services may change over time. Reliable evaluation requires reporting the exact model, visual input resolution, prompt, decoding settings, reasoning interface, and action representation. Comparisons with simpler semantic, geometric, and learning-based baselines are also necessary to determine whether the foundation model improves observation selection rather than only providing a stronger perception backbone.

#### 3.4.6. Hybrid and Distributed Decision Making

Many physical systems combine several decision mechanisms because no single method addresses semantic relevance, uncertainty, feasibility, long-term effects, and communication at the same time. A dual-resolution NBV planner for fusion reactor maintenance separates global low-resolution subregion selection from local high-resolution viewpoint selection, connecting large-scale mapping decisions with detailed observation planning [115]. A semantic model may identify a task-relevant region, a geometric module may generate reachable viewpoints, an information objective may rank them, and a motion planner may connect the selected view to the current platform state. A learned policy may estimate long-term value, while a distributed protocol determines which evidence should be shared. Semantic next best view planning, active localization and mapping, collaborative perception, and active manipulation commonly follow this form [16,26,39,54].

Hybrid systems can combine the transparency of explicit models with the flexibility of learned representations. Their main difficulty is attribution because several components may change simultaneously. An apparent improvement in active perception may result from a stronger perception backbone, a larger action space, a more accurate state representation, or a better controller rather than the sensing policy itself. Module level ablations and intermediate measurements are therefore necessary to show which part of the system improves the acquired evidence and which part improves the downstream task.

Distributed systems add another dependency because each agent acts on incomplete local information. The usefulness of a sensing action can depend on what other agents have observed, which messages have arrived, and whether shared information remains temporally valid. A robust distributed policy must continue operating under delayed or missing communication and should avoid relying on globally synchronized states that are unavailable during real deployment. The interpretation of these limitations also depends on the evaluation environment. Most representative methods summarized in Table 3 were evaluated primarily in simulated or benchmark environments, while physical-platform validation was reported only for a limited subset. The limitations supported by these evaluations therefore mainly concern computational scalability, model assumptions, training-data coverage, reward design, and simulated communication or interaction conditions. Potential sensitivity to calibration errors, sensor noise, control delay, dynamic occlusion, communication loss, reachability constraints, and safety risks should consequently be regarded as insufficiently validated deployment concerns unless they have been examined on physical platforms. The table therefore distinguishes limitations supported by the reported evaluation settings from the status of physical-platform validation.

### 3.5. Cross-Task Regularities in Active Observation Selection

The preceding analysis reveals several regularities that recur across reconstruction, localization, search, tracking, manipulation, and collaborative perception. These regularities do not depend on a particular task name or action selection algorithm. They arise from the relationship among the evidence required by the task, the action used to acquire that evidence, the predicted value of the resulting observation, and the physical process through which the action is executed.

First, observation value is meaningful only in relation to the state variable or task decision that requires additional evidence. Information gain is often presented as a general objective, but the uncertain quantity differs substantially across applications. Active recognition seeks observations that distinguish competing object hypotheses [18,45]. Active localization and mapping seek measurements that reduce uncertainty in the robot pose, map structure, or data association [16,27,31]. Active reconstruction seeks evidence about unobserved or poorly supported surfaces [13,14]. Collaborative perception seeks information that complements the receiving agent rather than information that is merely uncertain at the transmitting agent [26,35]. A numerical reduction in uncertainty therefore does not have a uniform interpretation. Its practical value depends on whether the reduced uncertainty concerns a variable that can change the next task decision.

Second, the action selection mechanism should not be confused with the objective of active perception. Reinforcement learning, probabilistic planning, geometric optimization, and foundation model reasoning describe procedures for selecting an action. They do not by themselves specify what evidence the task requires or why a particular observation is useful. Reinforcement learning can optimize surface coverage, localization quality, search success, target visibility, manipulation success, or communication efficiency according to its state and reward [13,60,111,113]. Foundation models can identify semantically relevant objects or regions, but this semantic relevance must still be connected to an observable visual condition and a task decision [3,37,38]. The capability of the action selection mechanism should therefore be evaluated separately from the perceptual objective that it is expected to serve.

Third, predicted observation value and physical executability are two different requirements. A candidate viewpoint may provide high geometric coverage or semantic relevance while remaining unreachable because of joint limits, obstacles, platform dynamics, or safety constraints. Sensor properties may also prevent a theoretically visible surface from producing a valid measurement [52]. This distinction is especially important in mobile manipulation, UAV inspection, and human centered sensing, where the camera pose changes the physical state of the platform and may interfere with the following task action [34,51]. A method should therefore report both the predicted benefit of a candidate observation and the feasibility, time, risk, and control error involved in acquiring it. Treating reachability as a final filtering step can lead the system to favor observations whose apparent value disappears after motion constraints are considered.

Fourth, final task performance alone cannot demonstrate that active observation selection was effective. A manipulation system may achieve higher success because it uses a stronger pose estimator or controller. A navigation policy may benefit from improved semantic representations even when its sensing actions provide little additional evidence. A collaborative system may improve detection by transmitting more features rather than selecting more complementary information. The contribution of active perception becomes clearer when evaluation measures the information deficit before the action, the direct change in observation quality after the action, the resulting change in task performance, and the resources used to obtain that change. Module level ablations are also required when the perception backbone, state representation, action space, controller, and sensing policy change simultaneously.

Fifth, fixed datasets cannot fully evaluate a closed-loop sensing policy when the policy is expected to change future observations. Prerecorded images and trajectories can test detection, depth estimation, localization, reconstruction, or observation value prediction under controlled inputs. They cannot determine whether another action would have produced a more useful observation because every policy receives the same predetermined evidence. The Active Vision Dataset provides a limited graph of selectable observations captured in real indoor environments [118]. Habitat and RoboTHOR allow an embodied policy to change its position and acquire different future views [119,120]. Physical platforms remain necessary when the research question involves reachability, actuation error, camera vibration, physical contact, safety, or communication delay. Closed-loop claims should therefore be supported by an environment in which the selected action actually changes the next observation.

A further regularity concerns the decision to stop sensing. Additional observations are not automatically beneficial. Once the current evidence is sufficient to support the task decision, another view can add motion, latency, computation, energy consumption, or physical risk without changing the outcome. Conversely, stopping too early leaves an unresolved information deficit. The stopping condition should therefore depend on the expected effect of another observation on the task rather than only on a fixed number of actions. This requirement connects evidence sufficiency, observation value, acquisition cost, and task progress within a single decision.

Together, these regularities show that active visual perception cannot be established merely by demonstrating that another viewpoint contains information or that an active policy achieves a higher final score. A convincing evaluation must show that the task required additional visual evidence, that the selected action could acquire that evidence under realistic constraints, that the new observation improved the relevant perceptual state, and that the resulting task benefit justified the acquisition cost. These requirements provide the basis for the evaluation principles discussed in Section 4.

## 4. Datasets, Platforms, Baselines, and Evaluation

Active visual perception cannot be evaluated through a single accuracy measure because the system changes the observations on which later perception and task decisions depend. A complete evaluation must determine whether the selected action produces better visual evidence, whether the new evidence improves the downstream task, and whether the improvement justifies the motion, computation, communication, energy, and safety costs required to obtain it. A method may achieve a more complete reconstruction while following an impractically long camera path. Another method may complete a task successfully because it uses a stronger perception backbone rather than a better sensing policy. Final accuracy or task success alone therefore cannot isolate the contribution of active observation selection.

The experimental requirements also differ across tasks. Active reconstruction requires geometric ground truth and controllable viewpoints. Active localization and exploration require an environment in which the current movement changes future observations. Search and tracking require targets whose visibility changes with camera or platform actions. Manipulation requires observation control to remain connected to reachability and physical task execution. Collaborative perception additionally requires controllable communication, relative pose error, delay, and information overlap. The following analysis distinguishes the resources used to support these experiments from the protocols used to judge the sensing decision.

### 4.1. Representative Datasets and Interactive Environments

Active reconstruction studies commonly use synthetic meshes, ShapeNet style object collections, Middlebury data, custom CAD models, and small collections of real objects [7,13,14,121]. These resources provide accurate camera poses, surface geometry, and depth ground truth. They make it possible to measure surface coverage, geometric error, reconstruction completeness, and the contribution of each additional view. However, many experiments select from predefined camera positions around an isolated object. Such settings test whether the policy can identify geometrically useful views, but they do not fully test whether those views are reachable by a physical robot.

Active localization and exploration use recorded trajectories, custom simulation maps, indoor environments, outdoor routes, and physical mobile robots [16,17,31,61]. KITTI, Freiburg, Intel Research Lab, and MIT Stata Center data can support evaluation of pose and map estimation. Their trajectories are fixed, however, which means that a policy cannot alter the route or acquire a different observation. They can therefore evaluate a localization or mapping component, but they cannot by themselves evaluate active route or viewpoint selection. Interactive simulation or a physical platform is required when the research question concerns where the robot should move, look, or revisit.

Search and navigation have a more developed set of shared interactive environments. The Active Vision Dataset provides dense RGB and depth observations at connected positions in real indoor scenes and supports controlled selection among neighbouring viewpoints [118]. Habitat provides efficient embodied simulation in three dimensional indoor environments [119], while RoboTHOR connects simulated scenes with corresponding physical environments for transfer evaluation [120]. These environments allow different policies to start from comparable states and change future observations through movement and gaze actions. Their results still depend on the available action set, object annotations, sensor model, and perception backbone.

Manipulation studies use tabletop simulation, custom object collections, physical manipulators, and general robot manipulation environments. Recent work has also introduced resources designed more directly for active observation. EFM10 evaluates exploratory and focused manipulation, while ActiveViewPose 200K and ActiveManip Bench evaluate active viewpoint acquisition and manipulation under changing visual evidence [39,66]. These resources move beyond the assumption that the robot begins from a favourable view. Their main difficulty is that final task performance remains jointly affected by perception, viewpoint selection, motion planning, and physical control.

Active tracking and collaborative perception often require custom interactive environments because fixed videos and fixed multiview datasets do not allow a policy to redirect a camera or reassign a sensor. Tracking studies therefore use controllable pan, tilt, and zoom cameras, camera networks, simulated moving targets, and physical video systems [5,6,104,122]. Collaborative driving studies use resources such as V2X-Sim, OPV2V, V2XSet, and DAIR-V2X to examine complementary observations and communication efficient fusion [26,35,36]. These resources provide multiview evidence, but some of them offer only limited control over how the next observations are generated. They are therefore more suitable for evaluating active message or region selection than for evaluating joint vehicle motion and observation control. Table 4 summarizes representative datasets and interactive environments together with their support for active evaluation and main limitations.

### 4.2. Simulation and Physical Platforms

Simulation provides repeatable interaction because different policies can begin from the same initial state while scene layout, target position, occlusion, sensor noise, and action budget remain controlled. It also supports the large number of trials required by reinforcement learning and foundation model-based policies. Embodied environments are suitable for search and navigation, robotics simulators support manipulation and exploration, and aerial or game engine environments can support UAV and active camera experiments. Their main advantage is not visual realism alone. Their essential property is that the selected action changes the next observation.

The action interface of the simulator strongly affects the result. A small discrete action space limits the number of possible viewpoints and makes search easier. Continuous movement introduces reachability, trajectory length, and control constraints. Simulated depth, pose, and visibility are often more reliable than physical measurements. Some environments also execute camera rotation or platform motion without realistic acceleration, blur, vibration, or delay. A policy may therefore appear efficient because the platform can acquire views that would be slow or unstable in a physical system.

Physical platforms reveal whether the selected observation can actually be obtained. Mobile robots expose wheel slip, localization drift, obstacle clearance, and limited camera placement. Manipulators introduce joint limits, self occlusion, collision, and coupling between the sensing pose and the following task action. UAVs introduce battery, wind, stability, and safety constraints. Camera networks introduce synchronization and calibration error, while multirobot systems additionally require relative localization and communication. These experiments provide stronger evidence of feasibility, but they are usually smaller and less repeatable than simulation studies.

Simulation and physical evaluation should therefore be interpreted together. Simulation can compare policies under controlled conditions, while physical experiments determine whether the predicted observation value survives sensor and execution error. A small physical demonstration cannot establish broad robustness, but simulation alone cannot establish that an informative viewpoint is reachable, timely, or safe.

### 4.3. Baselines and Evaluation Metrics

A passive or fixed observation baseline is required to determine whether changing the sensing process is necessary. This baseline keeps the viewpoint, route, sensor schedule, or communication policy unchanged. A random baseline tests whether the proposed action selection rule performs better than uninformed sampling. A uniform baseline distributes observations evenly and is especially relevant to reconstruction, inspection, and monitoring. A greedy baseline selects the action with the largest immediate value and helps determine whether longer horizon reasoning provides an additional benefit. When feasible, an oracle or privileged information baseline can estimate an upper bound, but its access to ground truth must be reported clearly.

Metrics should be reported at several levels. Observation level metrics measure what changed after the sensing action. Examples include target visibility, valid depth, surface coverage, entropy, pose covariance, detection confidence, and occlusion reduction. Task level metrics measure the effect on reconstruction, localization, search, tracking, manipulation, or collaboration. Examples include geometric error, ATE, RPE, search success, target loss, MOTA, IDF1, grasp success, and detection accuracy. Action level metrics measure the effort required to obtain the evidence, including view count, path length, camera rotation, action count, and completion time. System level metrics measure computation, memory, energy, communication volume, transmission delay, and safety violations.

These levels should not be replaced by a single weighted score. Two policies may achieve the same task success while using different travel distances or communication volumes. Two reconstruction methods may use the same number of views while producing very different camera paths. Reporting the underlying terms separately allows readers to determine whether an apparent gain remains useful under another platform or resource budget. Table 5 summarizes the main baselines and measurements required for closed-loop evaluation.

### 4.4. Requirements for Closed-Loop Evaluation

A fair comparison should keep the perception backbone, initial observation, candidate action space, sensing budget, and task controller consistent whenever the research question concerns the sensing policy. Otherwise, a higher final result may arise from a better detector, depth estimator, semantic model, or controller. When identical components cannot be used, the differences should be reported and supported through module level ablations.

Evaluation should also measure the state immediately before and after the sensing action. This comparison reveals whether the selected observation actually reduced occlusion, uncertainty, pose error, or another information deficit. Final task success remains important, but it should be reported together with this intermediate change. Without the intermediate measurement, the experiment cannot show whether the task improved because of better visual evidence or because another part of the system compensated for a weak sensing decision.

Privileged information must be identified explicitly. Ground truth maps, exact camera poses, perfect depth, known target locations, and complete communication graphs can be useful for isolating a planning component, but they are not available to a deployed system. Experiments should distinguish results obtained with estimated state from results obtained with ground truth state. Robustness tests should then introduce pose error, sensor noise, actuation delay, dynamic occlusion, packet loss, and unfamiliar environments according to the deployment setting.

Finally, the experiment should define when the system has acquired enough evidence. A fixed number of views makes comparison easier, but it does not show whether the policy can recognize that another observation is unnecessary. A stopping policy should be evaluated through the benefit of the final additional action, the cost of unnecessary observations, and the frequency with which the system stops before acquiring evidence needed by the task. This decision is part of active visual perception because sensing remains useful only while a new observation can change the subsequent estimate or action.

Existing resources demonstrate that active observation can improve reconstruction, localization, search, tracking, manipulation, and collaborative perception. However, no single dataset or platform currently provides common tasks, sensing actions, physical constraints, and resource measurements across all of these settings. The strongest evidence therefore comes from evaluations that combine shared interactive environments, controlled component comparisons, physical feasibility tests, and separate reporting of observation benefit, task improvement, and acquisition cost.

### 4.5. Applicability Boundaries of Datasets and Platforms

The applicability of each dataset or platform is determined not only by its scene domain and annotations, but also by whether it permits the sensing intervention claimed by the evaluated method. Synthetic meshes, CAD object collections, and densely sampled multiview datasets are suitable for evaluating geometric reconstruction, coverage, and next-best-view quality because they provide accurate geometry and controllable camera poses. Their applicability is limited when the research question involves reachability, collision avoidance, motion error, material-dependent sensing, or other physical constraints. Recorded trajectory datasets are suitable for evaluating localization, mapping, detection, and perception under realistic sensor observations. However, because their trajectories and observations are fixed, they cannot independently evaluate whether an active policy selects a better route, viewpoint, or sensing action.

Interactive simulators are suitable for closed-loop search, navigation, exploration, reinforcement learning, and long-horizon planning because an action can change the next observation under repeatable conditions. Their conclusions remain bounded by the fidelity of the action space, sensor model, dynamics, communication model, and visual domain. Real-world multiview, tracking, manipulation, and collaborative datasets provide realistic appearance, noise, occlusion, and communication conditions, but fixed camera layouts, predefined trajectories, or recorded messages may restrict evaluation to observation fusion, message selection, or perception rather than active motion control. Physical robot and camera platforms are required when the research claim concerns reachability, control delay, calibration, energy consumption, physical interaction, or safety. Nevertheless, physical experiments are usually platform-specific, costly, and less repeatable, and therefore cannot alone establish broad generalization. Dataset selection should consequently match the level of the research claim, and strong evidence should combine controlled simulation, realistic recorded data, and physical-platform validation whenever deployment feasibility is claimed.

### 4.6. Practical Checklist for Closed-Loop Evaluation

The preceding analysis can be consolidated into the following reusable checklist for designing and reporting closed-loop active visual perception experiments.

- **Closed-loop interaction.** The selected sensing action should actually change the subsequent observation, and the acquired evidence should update the state used for the next decision.
- **Appropriate evaluation setting.** The dataset, simulator, or physical platform should permit the sensing intervention claimed by the method and should match the intended level of the research claim.
- **Controlled comparison.** The initial observation, perception backbone, candidate action space, sensing budget, task controller, and stopping condition should be kept consistent whenever possible. Any unavoidable differences should be reported explicitly.
- **Baselines and ablations.** The method should be compared with appropriate passive or fixed, random, uniform, greedy, and, when feasible, oracle baselines. Component-level ablations should isolate the contributions of state estimation, observation-value prediction, action selection, and control.
- **Observation and task improvement.** Evaluation should report both the change in visual evidence before and after the sensing action and the resulting effect on the downstream task, rather than relying only on the final task score.
- **Cost, feasibility, and robustness.** View count, motion distance, completion time, computation, energy, communication, reachability, collision risk, and execution failures should be reported separately. Relevant sensor, state-estimation, execution, environmental, and communication disturbances should also be tested.
- **Information access.** Ground-truth states, exact poses, perfect depth, privileged maps, complete communication graphs, and any other information unavailable during deployment should be disclosed explicitly.
- **Stopping and reproducibility.** Both premature stopping and unnecessary additional observations should be measured. The number of trials, random seeds, failure criteria, system configurations, and sufficient action–observation traces should be reported to support reproduction.

## 5. Representative Applications

**Figure 4:**
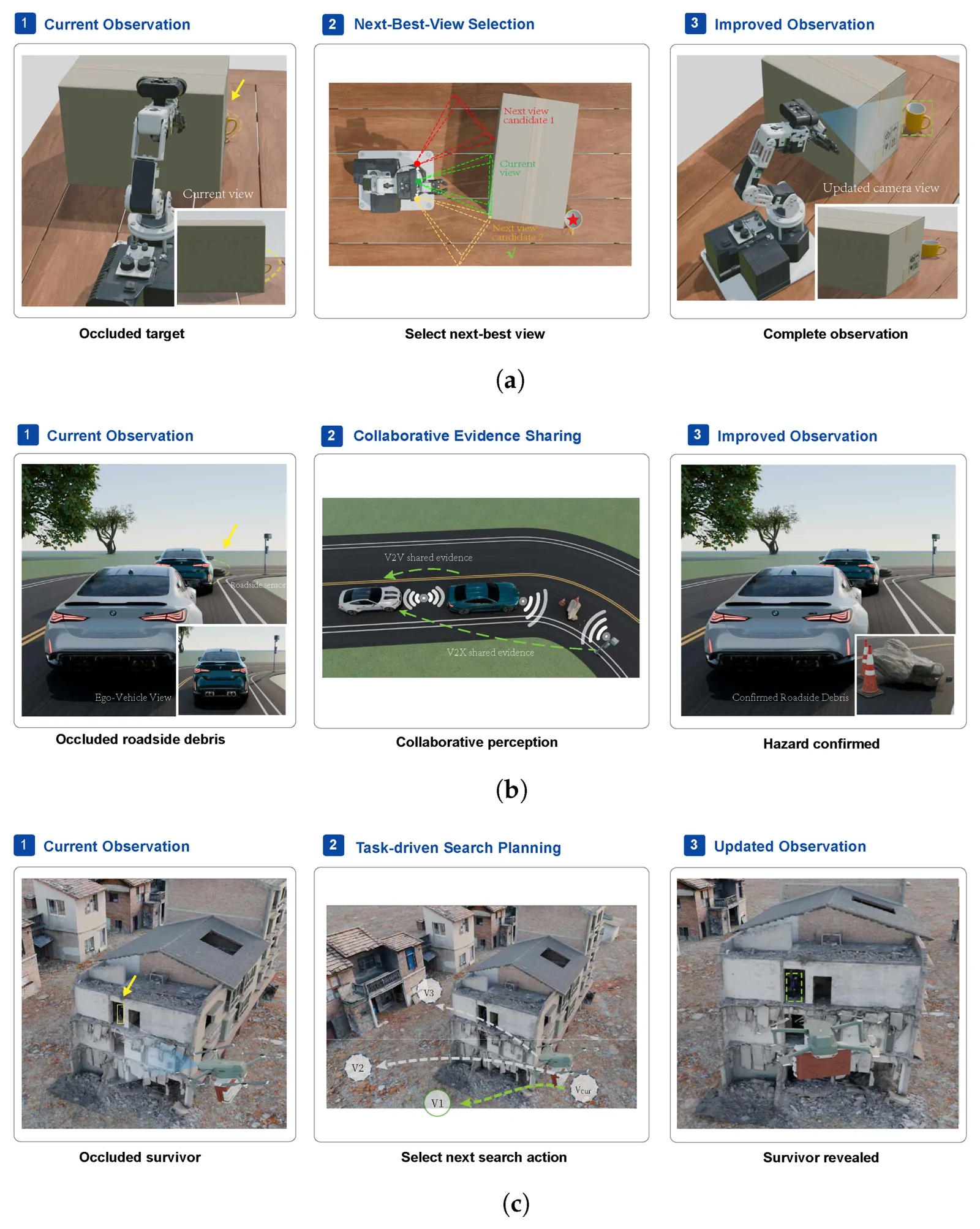
Representative active perception processes in robotic manipulation, collaborative driving, and UAV-assisted disaster rescue. In all subfigures, the yellow arrows point to the target, while the boxes in the lower-right corner of panels (**a**,**b**) show enlarged local views of the region observed from the current viewpoint. (**a**) Next-best-view selection for robotic manipulation, where the current and candidate viewpoints are distinguished by viewing cones with different colors; the asterisk marks the target, and the check mark indicates the selected viewpoint. (**b**) Collaborative driving, where the green dashed arrows indicate the sharing of complementary perceptual information between vehicles and between vehicles and the roadside sensor. (**c**) UAV-assisted disaster rescue, where the dashed arrows schematically illustrate three candidate search paths for acquiring task-relevant visual observations; the green dashed path indicates the selected search path.

The role of active visual perception changes with the task and the environment in which the system operates, as summarized in Table 6. Robotic manipulation uses additional viewpoints to reveal structures required for physical interaction. Autonomous vehicles and mobile robots seek observations that improve safety, localization, and decision-making in dynamic scenes. UAV systems adjust their flight and sensing strategies according to the urgency and spatial distribution of the task. Figure 4 illustrates three representative forms of active visual perception. These forms include next best view selection in robotic manipulation, complementary observation sharing in collaborative driving, and task-guided perception in UAV assisted disaster rescue.

### 5.1. Robotic Manipulation

In practical robotic manipulation, detecting an object does not necessarily provide the visual evidence required to act on it. In cluttered picking, the target may be recognizable while its graspable surfaces remain occluded, leaving the robot without a reliable collision-free grasp [123]. Similar difficulties arise when manipulation depends on an accurate object pose rather than object identity alone [124]. Transparent and specular objects further complicate deployment because their geometry may be poorly measured by depth sensors even when they are visible in an image [79]. The information requirement also changes during execution, as movement of the gripper or deformation of the object can alter what remains observable. Additional sensing must therefore be coordinated with the operation rather than treated as an independent preliminary stage. This creates an engineering tradeoff because moving the camera consumes time and workspace, while manipulating an occluder can change the scene that the robot must subsequently handle.

Existing methods address these difficulties by progressively expanding how the robot acquires missing evidence. Active vision systems adjust camera viewpoints to support manipulation, while visual servoing can guide observation toward less occluded regions [125,126]. Active pose estimation connects additional observations to object manipulation, and mobile manipulation extends this coordination to motion of the robot as a whole [34,124]. Probabilistic active grasping further links viewpoint selection to reconstruction uncertainty and online estimates of grasp success [123]. A concrete example of accounting for sensing cost is HEAPGrasp, which uses an RGB camera attached to the manipulator to reconstruct object geometry from silhouettes and balances measurement accuracy against camera travel. Its real robot experiments demonstrate grasping across different optical properties while reducing camera travel and handling time relative to its counterpart using a predefined trajectory around the scene. This illustrates how observation planning can improve acquisition efficiency rather than simply collect more views [79]. When camera motion alone is insufficient, interactive methods manipulate objects to improve detection, expose unobserved surfaces, or reveal hidden scene structure [22,23,63]. The Object-Aware Interactive Perception System implements selection between sensor repositioning and object manipulation on a dual-arm Baxter robot (Rethink Robotics, Boston, MA, USA) equipped with RGB and depth cameras [127]. Weng et al. extend coordinated observation and interaction to a deformable bag using two UR10e arms (Universal Robots A/S, Odense, Denmark), with one manipulating the bag and the other carrying a RealSense D435 camera (Intel Corporation, Santa Clara, CA, USA) [24]. Recent vision language action methods further incorporate observation adjustment into task execution [3,39].

Nevertheless, these methods remain constrained by the relationship between informative observations and executable manipulation. A useful camera pose must be reachable without compromising the subsequent operation, which makes coordinated perception and motion generation important in confined workspaces [34]. Avoiding unreliable depth sensing also does not eliminate perceptual assumptions, since HEAPGrasp still requires usable silhouette segmentation and camera pose information [79]. Interactive sensing adds another dependency because the action that reveals information changes the object state. In the bag study, the physical demonstration uses a restricted camera workspace and a visually marked opening, while separate simulation experiments report failures associated with excessive gripper motion and slower camera movement [24]. These conditions limit what the demonstrations establish about unrestricted deployment. Integrating observation and action within a learned policy does not by itself resolve such physical constraints. Engineering evaluation should therefore measure the complete sensing and manipulation cycle, distinguish improvements in visual evidence from changes in the perception or control model, and test recovery when an observation or interaction fails. The practical objective is to acquire enough evidence for reliable execution without allowing additional sensing to introduce disproportionate motion, delay, or scene disturbance.

### 5.2. Autonomous Driving and Mobile Navigation

Autonomous vehicles and mobile robots must make motion decisions before every relevant part of a changing environment can be observed. At an occluded intersection, onboard cameras and range sensors cannot determine whether a hidden region is empty or contains a pedestrian, cyclist, or vehicle about to enter the planned path. A planner that treats every hidden region as occupied may stop unnecessarily or become trapped, whereas a planner that ignores unobserved road users accepts an unmodelled collision risk. Visual navigation has a related evidence problem. Feature poor walls, doorway transitions, moving objects, illumination changes, and adverse weather can reduce stable visual correspondences and interrupt pose estimation even when the geometric route remains traversable [89,109,128]. The operational question is therefore whether the current observation supports the next motion decision with sufficient spatial accuracy and freshness.

Existing systems acquire complementary evidence through communication or controlled viewpoint changes. Learning-based collaborative perception methods such as Where2comm and How2comm select informative regions or communication partners instead of transmitting every observation [26,35]. In a simulation study of occluded driving, a V2X aware POMDP planner identifies hidden regions, represents possible unseen road users, selects external detections according to coverage, delay, and sensor reliability, and uses the selected evidence to update its belief before choosing a driving policy [89]. Engineering prototypes demonstrate parts of this process on physical systems. At the CETRAN autonomous vehicle test track, a roadside LiDAR system was connected to the Baidu Apollo autonomous driving platform (Baidu, Beijing, China) and Cohda Wireless communication hardware (Cohda Wireless Pty Ltd., Adelaide, Australia) under the SAE J2735 protocol. The experimental vehicle consequently received detections of vehicles and pedestrians before they entered the field of view of its onboard camera [129]. A more recent intelligent roadside unit integrates camera and LiDAR feature extraction on an FPGA with dual neural processing units and cellular V2X communication. Near sensor computation and selective transmission reduced end to end perception latency in real roadside trials and simulations [130]. Mobile platforms can instead change the observation itself. Semantic aware navigation for UAVs and outdoor active view planning for visual SLAM select routes or viewpoints that expose useful scene information [60,61], while adaptive camera view adjustment-based on the distribution of visual features has improved localization robustness in simulated and real environments [128].

These results do not yet establish a complete deployment chain from external observation to safety certified vehicle control. The CETRAN experiments were conducted on an enclosed test track, and the roadside detections were visualized and compared with onboard images rather than being used directly by the planning and control modules [129]. The V2X aware POMDP was evaluated in a proprietary simulator, while an asynchronous collaborative detection framework was evaluated on the V2X-Sim dataset rather than on communicating physical vehicles [89,131]. The latter study nevertheless exposes important deployment assumptions because exchanged detections still require a common time reference and accurate transformations between agents. Buildings can reduce packet delivery, communication delay can make moving object observations stale, and pose or calibration errors can place shared objects in the wrong part of the scene. Authors of active view planning research further report failures when learned policies encounter difficult transitions and insufficient visual texture, together with a need for human supervised training [109]. At the system level, camera actuation and viewpoint changing motion also consume time and energy and may conflict with collision avoidance or traffic constraints. Active perception is therefore useful only when expected information gain is balanced against communication, computation, calibration, and motion costs, and when the resulting observation remains valid when the downstream decision is executed.

### 5.3. Human Centered Interaction and Active Surveillance

Human centered robots and active surveillance systems must obtain task-relevant evidence about body pose, gaze, gesture, identity, and shared objects while people continue to move. Fixed cameras can lose hands, faces, or body parts because of self occlusion, restricted fields of view, and backlighting. In collaborative work, such missing evidence prevents a robot from reliably determining whether a person has entered its motion space or issued a gesture command. In surveillance, it can interrupt a trajectory and leave the identity or attributes of a target unresolved. The engineering problem is therefore not simply to detect a person, but to maintain sufficiently complete and timely observations for collision avoidance, interaction decisions, and target management [6,132].

Earlier studies established active visual search, gaze-based interfaces, and gesture interaction [133,134], while visual attention and proactive scene understanding allowed robots to redirect sensing as an interaction developed [135,136,137]. Recent engineering systems connect these principles more explicitly to physical sensing and camera motion. A multi-camera system dynamically captures RGB-D observations from important regions around a robotic arm and fuses partially visible body parts, which improves human sensing and supports obstacle avoidance under localized occlusion [132]. For active surveillance, a robotic camera array adjusts the positions and orientations of multiple cameras through cooperative reinforcement learning and has been evaluated for face detection and recognition in both simulation and real experiments [138]. Hybrid networks can also use fixed cameras for global person tracking and robot mounted cameras for close views that reveal task-specific attributes, although the reported evaluation of this arrangement remains photorealistic simulation [6]. These implementations extend earlier PTZ tracking and camera allocation methods [122,139,140,141]. A recent PTZ surveillance system further integrates image acquisition, object detection, and feedback control in a single processing loop and empirically examines how delayed observations and detector errors affect camera control [142].

These systems nevertheless remain constrained by the difference between an informative observation and an executable or acceptable one. Multi-view RGB-D sensing requires spatial calibration, synchronized observations, reliable association of body parts, and sufficient computation for fusion. Moving cameras additionally introduce actuator speed, mechanical rotation, communication delay, and control stability constraints. The PTZ study directly reports the effects of network latency, detection accuracy, and controller behaviour, while the hybrid mobile camera study is limited to simulation and should not be interpreted as a field deployment [6,142]. Privacy introduces another constraint because following a person or acquiring a closer view can capture information unrelated to the current task. A privacy-oriented alternative uses a 60 GHz industrial FMCW radar to estimate body pose and recognize gesture commands without relying on ordinary video images, but its evidence comes from a controlled dataset rather than closed-loop deployment in an operating production system [143]. A separate study using the Temi mobile robot (Robotemi Ltd., Tel Aviv, Israel) and the Ameca humanoid robot (Engineered Arts Ltd., Falmouth, UK) in a controlled care scenario shows that visible indications of sensor deactivation and the ability to move a robot away influence perceived control and trust [144]. At the system level, these findings imply that active observation policies must jointly consider perceptual benefit, calibration error, observation age, motion and computation costs, competition among targets, and the user’s ability to understand or restrict sensing.

### 5.4. UAV Search, Rescue, and Reconnaissance

UAV search, rescue, and reconnaissance systems must acquire observations that are geographically broad enough to locate relevant regions and sufficiently detailed to support an operational decision. High altitude images cover large areas but make people and other critical objects appear small against cluttered backgrounds, while lower flight provides more detail at the cost of a narrower footprint and greater exposure to obstacles. Forest canopies, damaged buildings, smoke, and poor illumination can prevent a top down camera from observing the ground, and the absence of reliable positioning makes an otherwise valid detection difficult to associate with a rescue location. Missing or poorly localized evidence can therefore prevent the system from confirming a possible victim, prioritizing an access route, or determining whether an area should be revisited [84,145,146].

Earlier UAV methods established semantic guidance, collaborative search, formation control, and multi-robot perception as mechanisms for directing sensing rather than applying uniform coverage alone [25,60,147,148]. Recent systems connect these ideas to more explicit engineering configurations. For forest search, a next best view planner evaluates how candidate camera positions and orientations expose surfaces that remain hidden by vegetation, and real environment experiments show that the selected oblique views improve observation beneath the canopy [84]. A heterogeneous rescue prototype combines UAVs for wide area observation with ground robots for closer inspection. RGB and thermal images are processed by FPGA-based edge hardware using a YOLO detector, allowing suspected survivors to be presented to rescuers without sending every image to a centralized computing platform [149]. Multi UAV systems can also use onboard cameras to estimate neighbouring vehicles, construct a time varying position belief field, and change their headings to reduce uncertainty or recover targets that have left the limited field of view. This architecture has been evaluated in both simulation and real flight experiments under communication denial [150]. For persistent reconnaissance, multiple quadrotors can lower their altitude above high priority regions to obtain higher resolution observations while maintaining wider coverage elsewhere, with distributed waypoint generation connecting changes in regional importance to subsequent flight decisions [151].

The available evidence nevertheless remains narrower than a mature emergency deployment. The forest next best view study includes real environment experiments with hidden objects, but it does not report an operational search for actual disaster victims. The heterogeneous UAV and ground robot system is a proof of concept whose components communicate through a shared wireless network, while the communication denied swarm experiments primarily demonstrate navigation, formation, and target recovery rather than a complete rescue mission [84,149,150]. A separate framework uses thermal sensing, LiDAR inertial localization, and belief-based planning to navigate under degraded visibility and unavailable satellite positioning, but its physical validation is a controlled indoor rescue style task rather than an outdoor disaster deployment [146]. Small person detection has also been demonstrated on embedded hardware and tested across different flight altitudes, yet this evidence comes mainly from image datasets and detector evaluation rather than a closed-loop autonomous search [145]. At the system level, these validation conditions imply that camera resolution, localization drift, calibration, communication loss, actuator response, wind, payload, battery capacity, and onboard computation can all change whether an informative viewpoint is reachable before its information becomes obsolete. Lowering altitude or revisiting an uncertain region may improve local evidence, but it consumes flight time and reduces the coverage available to the rest of the mission. Active observation policies for rescue and reconnaissance must therefore evaluate information gain together with flight feasibility, observation age, communication reliability, and the cost of leaving other regions unobserved.

### 5.5. Environmental Monitoring

Environmental monitoring must convert sparse, partially observed, and time varying signals into decisions about where to revisit, where to collect a physical sample, and when to report a meaningful change. In greenhouse crops, leaves and fruit clusters can hide the plant nodes required for harvesting and maintenance; so, a single image may not provide enough geometric evidence for manipulation. In lakes and reservoirs, measurements collected only at the surface or along a fixed route may miss localized water quality changes and their variation with depth. Repeated underwater surveys introduce a further difficulty because the system must distinguish an actual environmental change from differences caused by viewpoint, localization drift, illumination, or detection error. Fixed routes and uniform sampling therefore provide predictable coverage but cannot guarantee the target visibility, spatial confirmation, or temporal correspondence required for subsequent intervention [152,153,154] in Table 6.

Existing systems address these information deficits by using provisional observations to determine the next sensing action. Multimodal science mission planners can select both a motion path and a sensing modality according to their expected contribution under a limited mission budget [155]. Simulated plant inspection has similarly shown how local next best view planning can direct the camera toward occluded plant nodes [53]. This principle has subsequently been tested in an unmodified tomato greenhouse, where an RGBD camera collects observations from planned viewpoints, multiple object tracking associates plant nodes across views, and the resulting three dimensional semantic representation guides the next camera pose. The additional views reveal nodes hidden in earlier images and improve detection and localization relative to predefined viewpoints [152]. In aquatic monitoring, an Internet of Drones system combines water quality and depth sensors with wireless telemetry and a physical multisampler. Measurements of dissolved solids, depth, and estimated chlorophyll conditions can trigger additional sampling, while the accumulated observations support spatial mapping and depth profiling during field tests at a Lake Erie test site [153]. At a longer temporal scale, an autonomous underwater inspection framework uses semantic observations to refine its coverage path and compares maps from different sessions to revisit and localize possible changes. Its buoy monitoring experiments demonstrate how new observations can influence both the current route and the interpretation of later surveys [154]. Semantic route selection has also been studied for aerial robots in photorealistic simulation, where task-relevant objects rather than travel distance alone determine the preferred observation regions [60].

These systems also expose assumptions that become fragile on physical platforms. Information gain and semantic priority are useful only when the selected viewpoint is reachable, the sensor remains calibrated, and the new measurement can be registered consistently with earlier observations. Greenhouse systems must tolerate occlusion, natural illumination changes, sensor noise, camera pose uncertainty, and the motion time required to obtain supplementary views. Water monitoring must additionally account for sensor drift, delayed responses, depth dependent variation, communication coverage, payload capacity, and the energy consumed when an anomaly triggers further sampling. Multi robot source localization demonstrates that switching from broad coverage to concentrated active sensing can accelerate estimation, but its reported hardware in the loop and real time experiments use a radio source to emulate a gas field and therefore do not yet establish performance in a real pollutant plume [156]. Reactive benthic monitoring further shows that false detections can repeatedly divert an underactuated vehicle from area coverage. The proposed recovery mechanism was evaluated in simulation, while the associated field evidence comes from a reanalysis of earlier trials whose controller experienced false positives and navigation failures [157]. Environmental active perception must therefore evaluate observation value together with calibration reliability, registration error, onboard computation, communication, and the motion and energy costs of revisiting uncertain regions.

## 6. Key Challenges in Active Visual Perception

**Figure 5:**
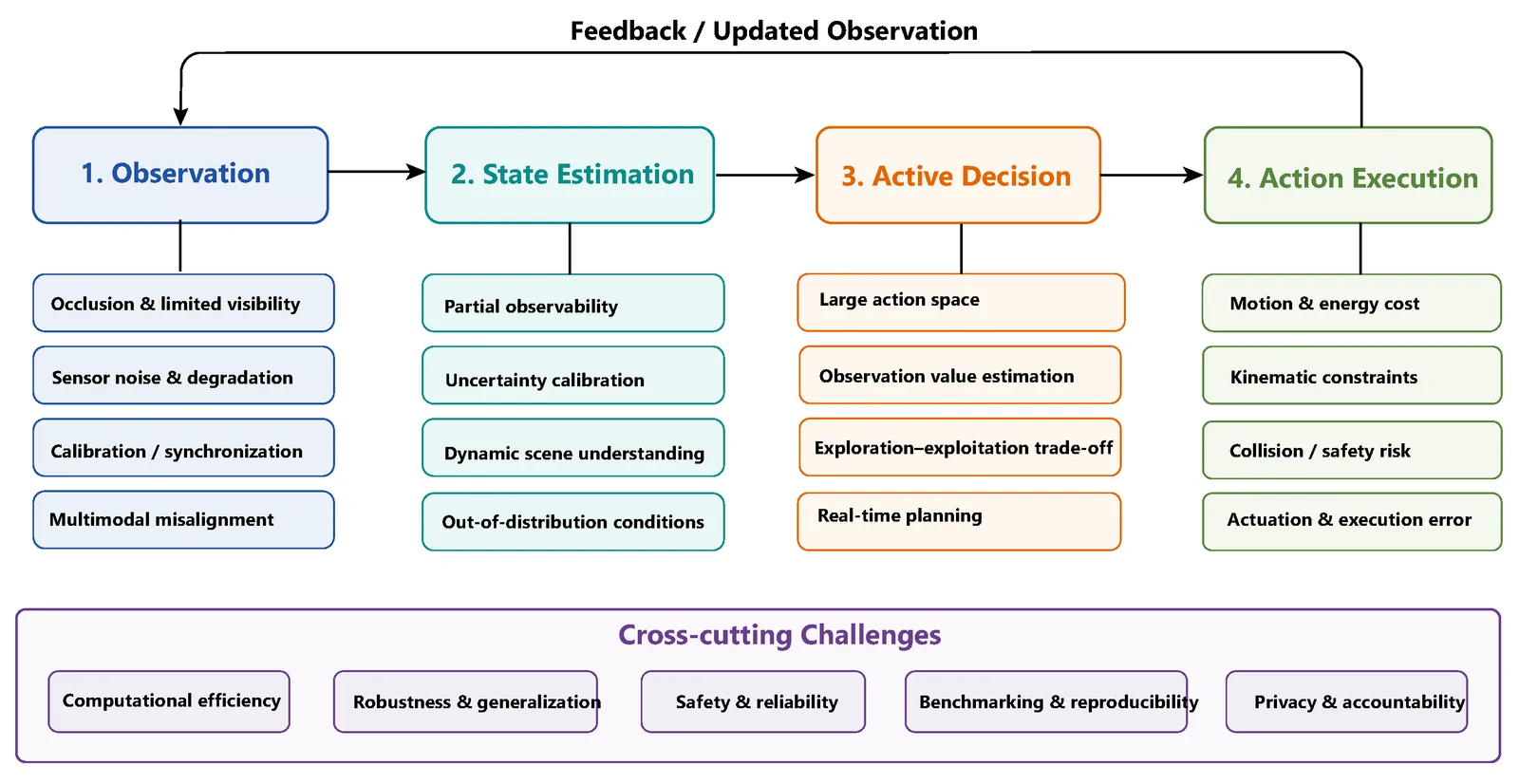
Interacting challenges in the active visual perception loop.

Figure 5 summarizes how these challenges interact within the active visual perception loop, while Table 7 compares their causes, consequences, and unresolved gaps. Active visual perception allows a system to improve its current information by changing the viewpoint, sensor configuration, or source of observation. This capability also gives perception errors more complex consequences. In passive vision, an incorrect estimate may affect only the current output. An active system can use the same error to select its next observation, causing later data to move further away from the evidence that is actually needed. View prediction, action execution, and state update are mutually dependent, and an error in one stage changes the visual evidence available to the stages that follow.

Existing methods can select informative viewpoints, plan exploration routes, and coordinate multiple sensors in controlled settings. Physical systems must nevertheless operate with incomplete observations, changing environments, motion constraints, limited computation, and sensor error at the same time. The central difficulty is no longer limited to finding an observation with high predicted value. The complete perception and action process must remain reliable under these conditions.

### 6.1. State Estimation in Incomplete and Changing Scenes

Active decisions first depend on how the system understands the current scene. The state must describe not only observed objects and space, but also which regions remain unknown, which estimates lack reliable evidence, and whether the remaining uncertainty affects the current task. A geometric map can represent unobserved space clearly, but it may not indicate whether that space is relevant to the task. A semantic map can highlight target objects and task-related regions, but incorrect labels can direct sensing toward the wrong location. A compact learned state supports rapid decisions, although it can make the visual evidence behind an action difficult to inspect.

The problem becomes more pronounced in open environments. Detectors and semantic models may appear reliable in familiar scenes while becoming overconfident under changing illumination, unfamiliar objects, or different language descriptions. Once an incorrect prediction is treated as a reliable state, the system may actively approach an irrelevant region and ignore the target that requires confirmation. Open vocabulary navigation has begun to examine uncertainty caused by prompt and model selection [20], but the wider connection between semantic confidence and reliable sensing actions remains unresolved.

Dynamic environments further shorten the period for which a state remains valid. People, vehicles, smoke, deformable objects, and other robots can move while the sensor is travelling toward the selected view. A viewpoint that appears useful at the time of planning may lose its value before execution. State estimation must therefore represent not only the current scene, but also target motion, changing occlusion, and the possibility that a prediction will become stale. Methods based on static maps and fixed candidate views become unreliable when environmental change approaches or exceeds the speed of action execution.

These factors affect the usefulness of a future observation through distinct but interacting mechanisms. Uncertainty in the current state makes the predicted benefit of a candidate view uncertain. Errors in estimated geometry, target position, or occlusion can change whether the required evidence will be visible. Prediction errors can then cause the system to overestimate how much that observation will reduce uncertainty or support the task. Delays between planning and image acquisition further increase the discrepancy between the predicted and actual observation, because targets, occluders, and the observing platform may move during this interval. Changes in the environment or task state can also make an accurately predicted observation less relevant to the decision that follows. Consequently, a viewpoint that appears informative when selected may provide little useful evidence when reached. Its value should therefore be assessed for the expected acquisition time and updated as new information becomes available, with replanning when the assumptions supporting the original selection no longer hold.

### 6.2. Action Selection Under Limited Time and Resources

The value of a single viewpoint can often be estimated from visibility, coverage, or expected uncertainty reduction. A sequence of sensing actions cannot be selected only from the next immediate gain. A locally informative viewpoint may move the robot away from the task goal or consume the time and energy required for later exploration. Conversely, an action with limited immediate benefit may expose a new region and place the system in a better position for later decisions. Active reconstruction, target search, and mobile exploration must therefore balance current observation value with future action opportunities. This balance should account not only for the expected benefit of an observation, but also for the time and energy required to reach and acquire it.

This dependence on future actions rapidly increases computational cost. The system must compare candidate observations, verify reachability, check safety, and determine whether the observation can be acquired in time. Continuous positions, camera orientations, imaging parameters, sensor choices, and communication actions further enlarge the search space. Greedy methods respond quickly, but they can repeatedly inspect a local region or consume the action budget too early. Longer planning horizons capture future consequences, although repeated observation prediction and motion checking can prevent real time execution.

Computational performance also extends beyond neural network inference. Candidate generation, visibility prediction, collision checking, communication delay, and actuator movement all affect when the new observation becomes available. In traffic and tracking, an accurate action can lose its value when it arrives too late. For example, a camera orientation selected to reveal a partially occluded target may become ineffective if the target moves behind another obstacle during computation or actuation. Evaluating observation utility should therefore account for the total time required for planning, communication, motion, and sensing, rather than assuming that the selected view is acquired immediately. Industrial inspection and offline reconstruction can afford more extensive planning. The system must also determine when to stop observing. Additional images only increase latency and resource use when they are unlikely to change the grasp, navigation, or search decision.

The stopping condition should therefore be task-dependent rather than defined only by a fixed number of views or an exhausted sensing budget. The system should continue observing only when the expected marginal benefit of another observation, such as a meaningful reduction in task-relevant uncertainty or a possible change in the downstream decision, justifies the associated time, energy, computation, and safety costs. Acquisition may stop successfully when the available evidence is sufficient for the task and the selected decision remains stable under plausible alternative observations. It may also stop because candidate views are expected to provide redundant information or because the remaining uncertainty cannot be resolved with the available viewpoints, sensing modalities, or time. In the latter case, stopping should not be interpreted as successful perception: the system may instead need to abstain, retreat, execute a conservative action, or request human assistance. Because the environment and task state can change, a stopping decision should also be revisited when new evidence invalidates the assumptions on which it was made.

### 6.3. From Informative Viewpoints to Executable Actions

Many methods first select an ideal camera pose in geometric space, but the physical platform determines whether that pose can be reached. A manipulator is limited by joint range and self occlusion, a mobile robot may not fit through the route that provides the desired view, and a UAV may be unable to remain stable near a structure. A viewpoint with high predicted value cannot produce useful evidence when it requires unsafe or infeasible motion.

Simulation often reduces these difficulties. Accurate depth, perfect calibration, and deterministic motion allow a policy to focus on viewpoint value, but they can also encourage dependence on information that a physical platform cannot provide. Motion blur, camera vibration, execution delay, and calibration drift affect both observation quality and action outcome. Randomization and fine tuning with real data can reduce part of this difference, but they do not replace explicit modelling of platform reachability, sensor placement, and execution error.

Accordingly, the utility of a future observation should be evaluated jointly with the cost, feasibility, and risk of obtaining it. A viewpoint with high expected information or task value may still be undesirable if reaching it requires excessive time or energy, violates the platform’s kinematic or dynamic constraints, or introduces substantial collision risk. Moreover, localization drift, control inaccuracies, actuator uncertainty, and external disturbances may cause the sensor to reach a pose different from the one assumed during planning, thereby reducing the quality or relevance of the acquired observation. Observation selection should therefore balance expected task benefit against acquisition time, energy consumption, motion feasibility, collision risk, and the probability and consequences of execution errors.

Robotic manipulation further shows that sensing actions and task actions cannot always be separated. Moving a wrist camera also changes the manipulator configuration, while rotating an object or removing an occluder changes the task state directly. Recent methods coordinate camera actions and manipulation actions within a shared process [3,39,93]. This integration reduces redundant movement between separate sensing and execution stages, but it also makes failure more difficult to diagnose. A failed manipulation may originate from viewpoint selection, state estimation, control error, or a mismatch among these components.

Errors at these stages do not remain isolated but can propagate through the closed perception–action loop. A sensing or state-estimation error may distort the predicted utility and feasibility of candidate viewpoints, causing the planner to select an uninformative, unsafe, or difficult-to-reach action. Errors during planning or motion execution may then cause the sensor to reach a pose different from the one assumed during observation prediction, changing the expected visibility, coverage, or information gain. If the resulting degraded observation is used to update the scene or target state without accounting for this mismatch, the estimation error can influence the next round of action selection and may be amplified over successive iterations. Conversely, uncertainty-aware state updating, execution monitoring, and replanning can interrupt this propagation and allow later observations to correct earlier errors. Robust active perception therefore requires uncertainty and failure information to be communicated across sensing, state estimation, observation prediction, action selection, and control, rather than treating errors at each stage independently.

### 6.4. Collaborative Perception Under Imperfect Communication

Multi agent systems can use sensors at different locations to reduce occlusion and extend the observed area, but collaboration does not simply require sharing more data. The agents must determine which member already possesses relevant information, which content is worth transmitting, and whether the remote observation will remain useful when it arrives. Redundant messages consume bandwidth, while missing a critical observation may cause several agents to enter the same unknown region.

Where2comm, How2comm, and ActFormer reduce communication through region or query selection [26,35,36]. These studies show that selective transmission can improve the balance between communication cost and perception quality. Even an accurate message can nevertheless change meaning because of transmission delay and pose error. A moving target may have left its reported location before the receiver uses the message, while inconsistent localization can prevent observations from different viewpoints from being aligned correctly.

As the number of agents grows, the system must jointly determine motion, observation regions, and communication relationships. Centralized policies can use global information, but their computational and communication costs increase rapidly. Independent decisions scale more easily, although they can produce repeated exploration, path conflicts, and observation gaps. Collaborative active perception must balance local autonomy with global coordination and retain basic task capability when communication becomes intermittent or an agent fails.

### 6.5. Safety, Privacy, and System Responsibility

Active visual perception obtains information through movement and interaction; so, its risks extend beyond incorrect recognition. A mobile robot may approach a person or obstacle to improve its view, a UAV may move closer to an unstable structure, and a manipulator may collide with or damage an object while revealing a hidden state. Observation gain does not by itself justify these actions, particularly when an additional view provides only a limited improvement.

Applications involving people must also consider whether the observation itself is acceptable. Camera redirection, persistent tracking, and local zoom can improve pose or identity recognition while collecting information unrelated to the task. A technically informative viewpoint may invade personal space, and a high resolution image may contain unnecessary sensitive content. The system must control not only where it observes, but also the spatial extent, duration, and later use of the observation.

Reliability also depends on whether the system can acknowledge insufficient evidence. An active policy may continue acquiring views when confidence remains unstable, or it may force a definite output when reliable evidence is unavailable. A safe system must distinguish uncertainty that can be resolved through another observation from uncertainty that cannot be resolved under the current conditions. Stopping, retreating, or requesting human assistance can be more appropriate than continuing when another observation is unlikely to change the decision or requires excessive risk.

### 6.6. Benchmarking, Reproducibility, and Fair Comparison

Existing active visual perception studies use substantially different scenes, action spaces, and resource constraints. Active reconstruction methods may be evaluated on different object collections and candidate viewpoints, while exploration methods use different map sizes, movement steps, and stopping conditions. Coverage, success, and path efficiency can therefore correspond to different levels of task difficulty, which makes direct comparison of final values misleading.

Fixed images and prerecorded trajectories provide consistent inputs for perception modules, but they cannot fully compare action selection because different policies cannot change future observations. Interactive simulation supports closed-loop comparison, although sensor models, action interfaces, and physical constraints also affect the result. The lack of corresponding simulation and physical settings makes it difficult to determine whether performance loss originates from the visual model, the sensing policy, or motion control.

Foundation models introduce additional reproducibility problems. Model versions, prompts, external services, and perception backbones can change over time. Replacing the semantic model may substantially alter the result even when the active decision component remains unchanged. Comparisons in active visual perception must therefore identify the available inputs, action permissions, model versions, and privileged information rather than recording only the policy name and final outcome.

These challenges do not occur independently. Incorrect uncertainty changes the predicted value of a viewpoint, computation delay makes the prediction stale, platform constraints prevent execution, and communication error alters the shared state. The central challenge is to maintain a reliable perception and action loop when these errors interact, rather than to select a high scoring next observation only under ideal conditions.

## 7. Future Directions

Active visual perception is moving from viewpoint control in constrained scenes toward intelligent systems that can adapt their sensing behavior to tasks, environments, and physical conditions. Future progress will be shaped by advances in task driven learning, multimodal sensing, embodied interaction, collaborative perception, and responsible deployment. These developments will allow active systems to acquire more relevant visual information, respond more effectively to environmental change, and operate with greater autonomy in complex real settings. At the same time, they will require closer coordination among perception, sensing actions, physical execution, and system safety.

### 7.1. Task Driven Learning and Foundation Models

Learning-based methods will continue to play an important role in active visual perception. Reinforcement learning allows a system to improve its sensing strategy through interaction, while self supervised and continual learning can reduce the dependence on manually labelled data. These methods are particularly useful when the relationship between a sensing action and its later task outcome is difficult to describe with a fixed geometric model. A robot can gradually learn which viewpoints support successful manipulation, while a mobile agent can learn which observations are most useful for navigation and target search.

Future research will place greater emphasis on observations that are directly related to the current task. A reconstruction system may not need to observe every surface when only a particular object part is required. A manipulation system may only need enough information to confirm a grasp region or an insertion point. Target-oriented reconstruction, affordance-guided viewpoint planning, and active manipulation already show this transition from generic coverage toward task-relevant evidence [39,51,54,69].

Foundation models will further extend this ability by connecting visual observations with open vocabulary concepts and language instructions. Systems such as ProFocus, Embodied Reasoner, ActiveVLA, and SaPaVe indicate that semantic reasoning can help identify which object, region, or task state requires additional observation [3,37,38,39]. However, these models may also produce plausible conclusions that are not supported by the current scene. Future systems will therefore need to distinguish prior knowledge from visual evidence and use new observations to verify uncertain semantic predictions rather than continuing from an unsupported assumption.

Here, an output produced by a foundation model, VLM, or VLA should be treated as a model-generated hypothesis derived from learned priors and the currently available inputs. It may describe an object, spatial relation, affordance, task state, or appropriate action even when the relevant scene evidence is occluded, ambiguous, outdated, or absent. By contrast, information that is observed and confirmed is grounded in measurements acquired by the sensing system from a task-relevant viewpoint and at the relevant time, and is supported by checks such as visibility, measurement quality, cross-view or cross-modal consistency, or agreement with the observed outcome of an executed action. Sensor confirmation does not imply error-free ground truth, but it provides direct and traceable evidence that a model-generated prediction alone cannot supply. Predictions from foundation models, VLMs, and VLAs should therefore guide what, where, and when to observe, while predictions that have not been verified should remain explicitly represented as uncertain hypotheses and should not be incorporated into the confirmed perceptual or task state until sufficient sensory evidence has been obtained.

### 7.2. Multimodal Sensing and Adaptive Sensor Control

Future active visual perception systems will rely on a wider range of sensing variables than camera position alone. Modern platforms may contain visual cameras, depth sensors, LiDAR, radar, thermal cameras, and other sensing devices. Each modality provides different information and performs differently under changes in distance, illumination, weather, and target motion. Cameras provide detailed appearance, LiDAR supports geometric measurement, radar remains effective under poor visibility, and thermal sensing can reveal people or objects in darkness.

Existing approaches illustrate different levels of spatial integration between learned perception models and sensor data. At the simpler end, Nowakowski et al. converted LiDAR point clouds into flat image-like representations so that pretrained image-based object detection and classification models could be reused, while the laser measurements supplied spatial information such as object distance [158]. This modular strategy can reduce the need to design a new perception model for each sensor representation, although projecting three-dimensional measurements into a flat view may discard geometric structure and introduce projection-dependent ambiguity. More advanced approaches construct a shared bird’s-eye-view representation. For example, BEVFormer v2 uses perspective supervision and a two-stage architecture in which proposals generated by a perspective-view head support final predictions in the BEV space, enabling modern image backbones to be adapted to multi-camera 3D detection [159]. These approaches span a progression from simplified reuse of pretrained image models on projected sensor data to learned spatial representations that support more explicit geometric reasoning. Within active perception, such fusion or representation mechanisms become observation-selection components when the system adaptively chooses the sensor, viewpoint, sampling configuration, or representation according to the current task and the evidence that remains uncertain.

The next stage of multimodal perception will involve more than combining all available sensor data. The system will need to decide which sensor should be used, when it should operate, and how its parameters should be adjusted. A platform may increase image resolution when a small object requires confirmation, raise the frame rate for a rapidly moving target, or switch to thermal sensing when visible images become unreliable. Multimodal scientific missions already show that observation value depends on both the sensing location and the selected modality [155].

Joint sensor control can improve robustness and reduce unnecessary computation, but it also introduces new difficulties. Zooming improves local detail while reducing the field of view. A higher frame rate provides better temporal information while increasing processing and energy use. Different sensors also require accurate spatial and temporal calibration before their observations can be combined. Future research will need hierarchical sensing policies that first identify the cause of insufficient information and then adjust only the viewpoint, imaging parameter, or sensing modality that is relevant to the problem.

### 7.3. Embodied Active Perception and Interaction

Active visual perception will become more closely integrated with the physical actions of robots. Many current systems still treat sensing as a stage that is completed before manipulation or navigation begins. This separation is difficult to maintain in real environments because every physical action can change the next observation. Moving a wrist camera changes the manipulator configuration, following a visually reliable route changes navigation cost, and touching or rotating an object changes both its appearance and its physical state.

Recent active manipulation and vision language action methods have begun to acquire new observations during task execution [3,39,93]. A robot can adjust its camera when a grasp region becomes occluded, inspect the result of an earlier action, or interact with an object when camera movement alone cannot reveal the required state. This continuous connection between sensing and acting can reduce unnecessary observation steps and improve adaptation to unexpected changes.

Physical interaction also introduces greater risk than camera movement. Rotating an object, opening a container, or removing an occluder may reveal useful information, but these actions can also disturb the scene or create an irreversible state change. Future systems will need to compare noncontact observation with physical interaction and select the safer option whenever both can provide sufficient evidence. They will also need to consider reachability, collision risk, recovery ability, and the effect of a sensing action on subsequent task execution.

### 7.4. Collaborative and Distributed Perception

Collaborative perception will continue to expand the sensing range of individual robots, vehicles, and UAVs. Different agents can observe the same environment from complementary locations and share information about targets that remain occluded or distant from one another. In autonomous driving, vehicles and roadside sensors can provide information beyond the line of sight of the ego vehicle. In disaster response, multiple UAVs can divide a search region and observe important locations from different directions.

Existing methods such as Where2comm, How2comm, and ActFormer have shown that selective communication can reduce data transmission while retaining useful perceptual information [26,35,36]. Future collaborative systems will need to move beyond deciding only what to transmit. They will also need to determine which agent should observe a target, where that agent should move, and whether the resulting information can still influence the current task decision.

Communication delay, pose error, and network failure will remain central obstacles. A message about a moving object may become outdated before it reaches another agent, while inaccurate relative poses may prevent observations from being aligned correctly. Larger teams also create repeated observations and conflicting movements when coordination is weak. Future distributed policies will need to preserve local autonomy, use communication only when it provides complementary evidence, and maintain basic task capability when the network becomes unreliable.

### 7.5. Open-World Adaptation and Long-Term Operation

Active visual perception systems deployed outside controlled experiments will encounter unfamiliar objects, new layouts, changing weather, sensor degradation, and different operating conditions. A sensing policy trained in one environment may continue selecting observations that were useful during training but no longer provide reliable evidence after the environment changes. Long-term operation therefore requires the system to recognize when its current perception and sensing strategy are no longer suitable.

Active sensing can support adaptation by directing the system toward observations that expose model errors or unfamiliar conditions. A robot may seek another viewpoint when an object does not match its existing representation, while an environmental monitoring system may revisit a region when the current appearance differs from its historical pattern. Continual learning can then update the perception model or sensing policy from these observations.

This process must remain controlled. An unfamiliar state should not automatically trigger aggressive exploration, particularly when physical interaction or movement may create risk. The system also needs to preserve earlier capabilities rather than repeatedly adapting to recent conditions and forgetting previous environments. Future research will need to connect uncertainty detection, safe exploration, continual learning, and long-term resource management so that adaptation improves the complete perception and action process rather than only the visual backbone.

### 7.6. Safety, Explainability, and Responsible Deployment

As active visual perception enters autonomous driving, surveillance, healthcare, human robot interaction, and other sensitive fields, sensing actions will need to satisfy physical, social, and ethical constraints. An active system does not only interpret information that has already been collected. It decides where to look, how closely to observe, and which information should be stored or shared. These decisions can affect personal space, privacy, safety, and public trust.

Future systems will need to limit sensing to the information required by the task. A camera should not continuously zoom toward a person when a lower resolution observation is already sufficient. A robot should not approach a person or fragile object when the expected information improvement is small. The system should also be able to stop, retreat, or request human assistance when uncertainty cannot be resolved safely.

Explainability will become especially important because an active decision changes which evidence is collected. The system should be able to indicate which information was missing, why a particular observation was selected, and whether the acquired evidence changed the decision. These explanations should be linked to the actual perceptual state rather than generated as general language descriptions. Transparent sensing records, privacy-preserving representations, human oversight, and clear responsibility boundaries will be necessary for dependable deployment.

Future progress in active visual perception will therefore depend on more than stronger perception models or a larger number of sensing actions. The field will need systems that understand which evidence matters to the task, select appropriate sensors and viewpoints, coordinate sensing with physical actions, cooperate under imperfect communication, adapt to unfamiliar environments, and operate within clear safety and ethical boundaries.

## 8. Conclusions

This review shows that active visual perception is not simply the processing of multiple observations, but a closed-loop process that connects task-related information needs, sensing actions, acquired evidence, and subsequent decisions. Across reconstruction, localization, search, tracking, manipulation, and multi-agent perception, a common finding is that an observation is useful only when it provides task-relevant evidence at an acceptable cost and can be acquired reliably under physical and environmental constraints.

The reviewed studies further show that uncertainty, delay, execution error, and environmental change can affect several stages of the perception–action loop, while multi-agent sensing requires coordination of who should observe, what should be communicated, and whether shared evidence remains timely and spatially aligned. These findings are supported by the cross-task comparisons in Section 3 and Section 5, the evaluation analysis in Section 4, and the recurring challenges summarized in Section 6. Future progress therefore requires closer integration of evidence verification, uncertainty-aware planning, executable sensing actions, adaptive stopping, and reproducible closed-loop evaluation.

## Figures and Tables

**Table 1:** Comparison between representative previous reviews and the analytical scope of this review.

Review	Primary Scope	Observation Control	Decision Emphasis	Evaluation/Constraints
Bajcsy et al. [1]	Conceptual foundations of active perception	What, where, when, and how to sense	Task and context dependent sensing	Resource constraints discussed conceptually
Chen et al. [8]	Active vision in robotic systems	Sensor pose, configuration, and viewpoint planning	Uncertainty and task-oriented sensor planning	Robot configurations, applications, and method evaluation
Zeng et al. [30]	View planning for robotic active vision	Primarily sensor viewpoint and view sequence	View quality and planning strategies	Hardware conditions, scanning state, and planning efficiency
Ahmed et al. [31]	Active and collaborative SLAM	Robot trajectory and control action selection	Pose and map uncertainty, information utility	Planning methods, deployment settings, and SLAM performance
**This review**	**Cross-task active observation acquisition**	**Viewpoint, gaze, sensing, interaction, and coordination**	**Required evidence, observation value, and action selection**	**Evidence improvement, task improvement, feasibility, and resource cost**

**Table 2:** Literature areas and representative search terms used in this review.

Literature Area	Representative Search Terms
Foundations and prior reviews	Active vision; active visual perception; active sensing; revisiting active perception
Next best view and active reconstruction	Next best view; active 3D reconstruction; active view planning; viewpoint selection for reconstruction
Active SLAM and exploration	Active SLAM; active visual SLAM; information gain for active SLAM; semantic active SLAM; active exploration
Visual search, recognition, and navigation	Active visual search; embodied visual search; active object recognition; active perception for object navigation
Camera control, gaze, and tracking	Active camera control; pan tilt zoom camera tracking; gaze control; active viewpoint tracking; multi-camera tracking
Robotic manipulation and interactive perception	Active perception for robotic manipulation; manipulation under occlusion; interactive perception; active viewpoint selection for manipulation
Collaborative perception and sensor selection	Collaborative active perception; multi-agent active perception; multi-robot exploration; cooperative next best view; active sensor selection

Note: The listed terms represent the main search directions used to identify relevant literature rather than an exhaustive set of fixed search queries. Additional studies were identified through backward and forward citation tracing from foundational reviews and representative recent work.

**Table 3:** Comparison of representative mechanisms for selecting sensing actions in active visual perception. The reported simulation limitations and the status of physical-platform validation are distinguished.

Decision Mechanism and Representative Studies	Required Information	Suitable Sensing Decisions	Evaluation Setting and Main Limitation
Rule-based and geometric [7,21,52,58]	Designed states, geometric models, visibility, and reachability	Gaze control, camera pose, viewpoint, and platform motion	*Simulation evidence.* Evaluation is limited to simulation, where performance depends on designed rules, geometric accuracy, and idealized visibility. *Physical-platform validation.* Not reported; robustness to calibration errors, dynamic occlusion, material effects, and actuation uncertainty remains unverified.
Probabilistic and belief-based [18,116,117]	State beliefs, observation likelihoods, and uncertainty models	Viewpoint, revisit action, path, and sensing sequence	*Simulation evidence.* Evaluation is limited to simulation, where computation increases with belief dimensionality and planning horizon and depends on assumed noise models. *Physical-platform validation.* Not reported; robustness to sensor failures, likelihood miscalibration, measurement noise, and real-time constraints remains unverified.
Information-based optimization [27,98,99]	Calibrated state uncertainty and predicted observation updates	Viewpoint, waypoint, sensor, and measurement selection	*Simulation evidence.* Evaluation is limited to simulation, where observation values inherit errors from idealized belief and sensor models. *Physical-platform validation.* Not reported; whether predicted information gain is preserved under sensor noise, model mismatch, and execution delay remains unverified.
Search and receding horizon planning [15,19,61,106]	Candidate actions, transition models, and predicted future observations	View sequences, exploration routes, and continuous camera motion	*Simulation evidence.* Evaluation is limited to simulation, where computation grows with the action space and prediction depth and future observations are often idealized. *Physical-platform validation.* Not reported; robustness to planning latency, motion uncertainty, dynamic obstacles, and reachability constraints remains unverified.
Supervised and imitation learning [2,14,49]	View labels, expert values, or demonstrations	Viewpoint, gaze, zoom, and camera behavior	*Simulation evidence.* Evaluation is limited to simulation, where policies inherit training-data coverage limits and may overfit simulated appearance or action discretization. *Physical-platform validation.* Not reported; robustness to changes in lighting, camera calibration, motion blur, and actuator response remains unverified.
Reinforcement learning [13,60,111,113]	Interaction experience, rewards, and environment dynamics	Path, viewpoint, gaze, and task coupled sensing actions	*Simulation evidence.* Evaluation is limited to simulation, where policies remain sensitive to reward design and may exploit simulator-specific appearance or dynamics. *Physical-platform validation.* Not reported; sim-to-real transfer, safe exploration, control delay, and reliability under physical disturbances remain unverified.
Foundation model-based reasoning [3,20,37,38]	Language instructions, semantic representations, and reasoning history	Semantic queries, relevant regions, viewpoints, and embodied actions	*Simulation evidence.* Evaluation is limited to simulated or benchmark environments, where semantic confidence may be mistaken for visual evidence and results remain sensitive to prompts and model versions. *Physical-platform validation.* Not reported; real-time visual grounding, action reachability, inference latency, and safe closed-loop execution remain unverified.
Collaborative and distributed [6,26,36,46]	Local observations, agent states, message value, and communication status	Agent assignment, viewpoint coordination, and message selection	*Simulation evidence.* Evaluation is limited to simulation, where communication, synchronization, and pose information are typically simplified. *Physical-platform validation.* Not reported; robustness to packet loss, variable delay, limited bandwidth, pose error, and temporal misalignment remains unverified.
Interactive and hybrid [22,23,24,39,54]	Task state, physical constraints, semantic relevance, and predicted outcomes	Physical interaction and combinations of sensing, motion, and communication actions	*Simulation evidence.* Evaluation in simulated settings shows that module coupling and idealized interaction models can make performance attribution difficult. *Physical-platform validation.* Reported in at least one representative study; physical robot experiments expose practical constraints including restricted camera workspaces and interaction uncertainty. Broader robustness to hardware variation, unrestricted environments, and physical safety remains insufficiently validated.

Note. “Simulation evidence” denotes limitations supported by the reported simulation or benchmark experiments. “Physical-platform validation not reported” indicates that the corresponding deployment concerns have not been experimentally demonstrated on a physical real-world platform.

**Table 4:** Representative datasets and interactive environments used in active visual perception.

Task	Representative Resources	Support for Active Evaluation	Main Limitation
Active reconstruction [7,13,14,121]	Synthetic meshes, ShapeNet style objects, Middlebury data, custom CAD models, and real object collections	Accurate geometry and controllable views support coverage, completeness, and view contribution analysis	Viewpoints are often predefined and ignore reachability, collision, and motion error
Active localization and exploration [16,17,31]	KITTI, Freiburg, Intel Research Lab, MIT Stata Center, custom simulators, and mobile robots	Interactive settings support route, revisit, coverage, and loop closure decisions	Recorded trajectories cannot evaluate action selection, while custom environments limit direct comparison
Search and navigation [118,119,120]	Active Vision Dataset, Habitat, RoboTHOR, and related indoor embodied environments	Policies can change position and gaze and acquire task dependent observations	Results depend on annotations, action discretization, sensor assumptions, and the perception backbone
Active tracking [6,104,122]	Controllable camera networks, pan tilt zoom systems, simulated targets, and physical video platforms	Camera orientation, assignment, and target handover can affect future visibility	Custom target dynamics and camera models reduce comparability across studies
Robotic manipulation [34,39,66]	Tabletop simulation, physical manipulators, EFM10, ActiveViewPose 200K, and ActiveManip Bench	Viewpoint and interaction actions can be evaluated together with task execution	Perception, sensing, planning, and control gains remain difficult to separate
Collaborative perception [26,35,36,99]	V2X-Sim, OPV2V, V2XSet, DAIR-V2X, multirobot maps, and camera networks	Supports complementary observation, message selection, and communication cost analysis	Accurate poses, synchronization, and stable communication can overestimate collaborative gains

**Table 5:** Baselines and measurements required for closed loop evaluation of active visual perception.

Evaluation Question	Required Baseline or Measurement	What It Reveals	Common Confounding Factor
Is active sensing necessary?	Passive or fixed observation baseline	Whether changing the observation improves perception or the downstream task	The active method may also use a stronger perception model
Is the selection rule informative?	Random and uniform action baselines	Whether task or uncertainty information improves action choice	Different candidate action sets may favour one method
Does planning horizon matter?	Greedy or immediate utility baseline	Whether reasoning about later observations improves the result	Longer planning may use more computation or privileged prediction
Did the observation itself improve?	Visibility, coverage, uncertainty, valid depth, or pose quality before and after the action	The direct contribution of the acquired evidence	The metric may be poorly calibrated or unrelated to the task
Did the task improve?	Reconstruction, localization, search, tracking, manipulation, or detection performance	Whether the new evidence changes the final task outcome	Control quality and the perception backbone also affect the result
Was the improvement efficient?	View count, path length, action count, time, energy, computation, and communication	The resources required to obtain the reported improvement	A combined score can hide which resource caused the difference
Is the policy robust?	Sensor noise, pose error, actuation delay, packet loss, dynamic occlusion, and unseen scenes	Whether the sensing decision remains useful outside ideal settings	Perturbation ranges and failure definitions differ across studies
Did the system stop appropriately?	Marginal gain, additional action benefit, and stopping error	Whether further sensing would change the task decision	Many studies use a fixed action budget instead of a learned stop rule

**Table 6:** Perception–action characteristics of representative application settings.

Application	Information Deficit	Controlled Action	Expected Evidence	Dominant Constraints
Robotic manipulation	Hidden grasp region, uncertain pose, or unknown object state	Wrist or head motion, base motion, active-view arm, or object interaction	Clearer affordance, improved pose, or interaction-generated state evidence	Collision, reachability, irreversible action, latency, and task coupling
Autonomous driving	Occluded road user, weak visibility, or incomplete local sensing	Camera orientation, adaptive resolution, modality selection, or collaborative query	Target confirmation, complementary geometry, or remote hazard evidence	Real-time response, safety, weather, synchronization, and pose error
Human-centered systems	Occluded body, uncertain gesture, or missing shared-task evidence	Gaze, camera orientation, robot repositioning, or selective resolution	Clearer pose, attention, intention, or shared-object state	Personal space, privacy, interpretability, consent, and social acceptance
Surveillance and security	Blind spot, target exit, identity ambiguity, or limited resolution	Pan, tilt, zoom, camera assignment, or target handover	Centered target, identity-preserving sequence, or complementary views	Target competition, control delay, privacy, and operator accountability
UAV search and rescue	Unknown layout, hidden victim, or inaccessible region	Flight path, altitude, orientation, revisit, or team allocation	Victim evidence, damage map, route information, or wide-area coverage	Battery, collision, urgency, wind, and communication
Environmental monitoring	Sparse anomaly, changing conditions, or uneven target distribution	Route, viewpoint, sensor schedule, or spatially varying sampling	Focused anomaly evidence and long-term change observations	Energy, weather, revisit fairness, and long-term autonomy

**Table 7:** Main challenges and unresolved gaps in active visual perception.

Challenge	Why It Arises	Consequence for Active Perception	Unresolved Gap
State estimation in incomplete and changing scenes	Partial observations, semantic errors, poorly calibrated confidence, and changing occlusion	The system may select actions from an incorrect or outdated state and reinforce its initial error	State representations that preserve task relevance, temporal validity, and calibrated geometric and semantic uncertainty
Action selection under limited time and resources	Long-term action dependence, large action spaces, and expensive prediction and feasibility checking	Greedy decisions can waste the sensing budget, while more extensive planning may become too slow for the task	Planning methods that adapt their horizon and computational effort to task urgency and available resources
Executable sensing on physical platforms	Ideal viewpoints may ignore reachability, embodiment, motion error, and interference with the task action	An informative viewpoint may be unreachable, unsafe, or detrimental to the subsequent operation	Joint reasoning about observation value, platform dynamics, task feasibility, and transfer from simulation to physical systems
Collaborative perception under imperfect communication	Limited bandwidth, transmission delay, pose disagreement, and agent or sensor failure	Shared evidence may become outdated or misaligned, while poor coordination causes redundant sensing or observation gaps	Distributed policies that coordinate motion, sensing, and message selection while remaining reliable under intermittent communication
Safety, privacy, and system responsibility	Active movement, physical interaction, camera redirection, zoom, and persistent observation can affect people and the environment	The system may cause collision, object damage, privacy intrusion, or unjustified actions under unresolved uncertainty	Risk aware stopping, minimum necessary observation, human oversight, and interpretable reasons for sensing actions
Benchmarking, reproducibility, and fair comparison	Studies use different scenes, action spaces, sensing budgets, perception backbones, and model versions	Reported results are difficult to compare, and the source of a performance gain or failure often remains unclear	Shared closed-loop settings, transparent system configurations, and corresponding simulation and physical evaluations

## Data Availability

No new data were created or analyzed in this study. Data sharing is not applicable to this article.

## References

[1] Bajcsy R., Aloimonos Y., Tsotsos J.K. (2018). Revisiting active perception. Auton. Robot..

[2] Xiong H., Xu X., Wu J., Hou Y., Bohg J., Song S. Vision in Action: Learning Active Perception from Human Demonstrations. Proceedings of the 9th Annual Conference on Robot Learning.

[3] Liu Z., Gu Y., Wang Y., Xue X., Fu Y. (2026). ActiveVLA: Injecting active perception into vision-language-action models for precise 3D robotic manipulation. arXiv.

[4] Xu R., Xia X., Li J., Li H., Zhang S., Tu Z., Meng Z., Xiang H., Dong X., Song R. (2023). V2v4real: A real-world large-scale dataset for vehicle-to-vehicle cooperative perception. Proceedings of the 2023 IEEE/CVF Conference on Computer Vision and Pattern Recognition (CVPR).

[5] Murray D., Basu A. (1994). Motion tracking with an active camera. IEEE Trans. Pattern Anal. Mach. Intell..

[6] Casao S., Serra-Gómez Á., Murillo A.C., Böhmer W., Alonso-Mora J., Montijano E. (2024). Distributed multi-target tracking and active perception with mobile camera networks. Comput. Vis. Image Underst..

[7] Dunn E., Frahm J.M. Next Best View Planning for Active Model Improvement. Proceedings of the BMVC.

[8] Chen S., Li Y., Kwok N.M. (2011). Active vision in robotic systems: A survey of recent developments. Int. J. Robot. Res..

[9] Bajcsy R. (1988). Active perception. IEEE.

[10] Aloimonos J., Weiss I., Bandyopadhyay A. (1988). Active vision. Int. J. Comput. Vis..

[11] Ballard D.H. (1991). Animate vision. Artif. Intell..

[12] Swain M.J., Stricker M.A. (1993). Promising directions in active vision. Int. J. Comput. Vis..

[13] Peralta D., Casimiro J., Nilles A.M., Aguilar J.A., Atienza R., Cajote R. (2020). Next-best view policy for 3d reconstruction. Proceedings of the European Conference on Computer Vision.

[14] Chen X., Li Q., Wang T., Xue T., Pang J. (2024). Gennbv: Generalizable next-best-view policy for active 3d reconstruction. Proceedings of the 2024 IEEE/CVF Conference on Computer Vision and Pattern Recognition (CVPR).

[15] Leung C., Huang S., Dissanayake G. (2006). Active SLAM using model predictive control and attractor based exploration. Proceedings of the 2006 IEEE/RSJ International Conference on Intelligent Robots and Systems.

[16] Placed J.A., Rodríguez J.J.G., Tardós J.D., Castellanos J.A. (2022). Explorb-slam: Active visual slam exploiting the pose-graph topology. Proceedings of the Iberian Robotics Conference.

[17] Bonetto E., Goldschmid P., Pabst M., Black M.J., Ahmad A. (2022). irotate: Active visual slam for omnidirectional robots. Robot. Auton. Syst..

[18] Laporte C., Arbel T. (2006). Efficient discriminant viewpoint selection for active bayesian recognition. Int. J. Comput. Vis..

[19] Taioli F., Giuliari F., Wang Y., Berra R., Castellini A., Del Bue A., Farinelli A., Cristani M., Setti F. (2024). Unsupervised active visual search with monte carlo planning under uncertain detections. IEEE Trans. Pattern Anal. Mach. Intell..

[20] Bajpai U., Rückin J., Stachniss C., Popović M. (2025). Uncertainty-informed active perception for open vocabulary object goal navigation. Proceedings of the 2025 European Conference on Mobile Robots (ECMR).

[21] Rivlin E., Rotstein H. (2000). Control of a camera for active vision: Foveal vision, smooth tracking and saccade. Int. J. Comput. Vis..

[22] Le Q.V., Saxena A., Ng A.Y. (2010). Active Perception: Interactive Manipulation for Improving Object Detection.

[23] Krainin M., Curless B., Fox D. (2011). Autonomous generation of complete 3D object models using next best view manipulation planning. Proceedings of the 2011 IEEE International Conference on Robotics and Automation.

[24] Weng Z., Zhou P., Yin H., Kravberg A., Varava A., Navarro-Alarcon D., Kragic D. (2024). Interactive perception for deformable object manipulation. IEEE Robot. Autom. Lett..

[25] Queralta J.P., Taipalmaa J., Pullinen B.C., Sarker V.K., Gia T.N., Tenhunen H., Gabbouj M., Raitoharju J., Westerlund T. (2020). Collaborative multi-robot search and rescue: Planning, coordination, perception, and active vision. IEEE Access.

[26] Hu Y., Fang S., Lei Z., Zhong Y., Chen S. (2022). Where2comm: Communication-efficient collaborative perception via spatial confidence maps. Adv. Neural Inf. Process. Syst..

[27] Mu B., Giamou M., Paull L., Agha-mohammadi A.a., Leonard J., How J. (2016). Information-based active SLAM via topological feature graphs. Proceedings of the 2016 IEEE 55th Conference on Decision and Control (CDC).

[28] Tarabanis K.A., Allen P.K., Tsai R.Y. (1995). A survey of sensor planning in computer vision. IEEE Trans. Robot. Autom..

[29] Scott W.R., Roth G., Rivest J.F. (2003). View planning for automated three-dimensional object reconstruction and inspection. ACM Comput. Surv. (CSUR).

[30] Zeng R., Wen Y., Zhao W., Liu Y.J. (2020). View planning in robot active vision: A survey of systems, algorithms, and applications. Comput. Vis. Media.

[31] Ahmed M.F., Masood K., Fremont V., Fantoni I. (2023). Active slam: A review on last decade. Sensors.

[32] Placed J.A., Strader J., Carrillo H., Atanasov N., Indelman V., Carlone L., Castellanos J.A. (2023). A survey on active simultaneous localization and mapping: State of the art and new frontiers. IEEE Trans. Robot..

[33] Maboudi M., Homaei M., Song S., Malihi S., Saadatseresht M., Gerke M. (2023). A review on viewpoints and path planning for UAV-based 3-D reconstruction. IEEE J. Sel. Top. Appl. Earth Obs. Remote Sens..

[34] Jauhri S., Lueth S., Chalvatzaki G. (2024). Active-perceptive motion generation for mobile manipulation. Proceedings of the 2024 IEEE International Conference on Robotics and Automation (ICRA).

[35] Yang D., Yang K., Wang Y., Liu J., Xu Z., Yin R., Zhai P., Zhang L. (2023). How2comm: Communication-efficient and collaboration-pragmatic multi-agent perception. Adv. Neural Inf. Process. Syst..

[36] Huang S., Zhang J., Li Y., Feng C. (2024). Actformer: Scalable collaborative perception via active queries. Proceedings of the 2024 IEEE International Conference on Robotics and Automation (ICRA).

[37] Xue W., Li M., Wu X., Tang J., Yang D., Zhang L. (2026). ProFocus: Proactive Perception and Focused Reasoning in Vision-and-Language Navigation. arXiv.

[38] Zhang W., Wang M., Liu G., Xu H., Jiang Y., Shen Y., Hou G., Zheng Z., Zhang H., Li X. (2026). Embodied-Reasoner: Synergizing visual search, reasoning, and action for embodied interactive tasks. Proceedings of the 64th Annual Meeting of the Association for Computational Linguistics (Volume 1: Long Papers).

[39] Liu M., Zhou E., Chi C., Han Y., Rong S., Chen L., Wang P., Wang Z., Zhang S. (2026). Sapave: Towards active perception and manipulation in vision-language-action models for robotics. arXiv.

[40] Liu H., Guo D., Cangelosi A. (2025). Embodied intelligence: A synergy of morphology, action, perception and learning. ACM Comput. Surv..

[41] Vasquez-Gomez J.I., Sucar L.E., Murrieta-Cid R., Lopez-Damian E. (2014). Volumetric next-best-view planning for 3D object reconstruction with positioning error. Int. J. Adv. Robot. Syst..

[42] Feng Z., Zhan H., Chen Z., Yan Q., Xu X., Cai C., Li B., Zhu Q., Xu Y. (2024). Naruto: Neural active reconstruction from uncertain target observations. Proceedings of the 2024 IEEE/CVF Conference on Computer Vision and Pattern Recognition (CVPR).

[43] Chen L., Zhan H., Chen K., Xu X., Yan Q., Cai C., Xu Y. (2025). Activegamer: Active gaussian mapping through efficient rendering. Proceedings of the 2025 IEEE/CVF Conference on Computer Vision and Pattern Recognition (CVPR).

[44] Forssén P.E., Meger D., Lai K., Helmer S., Little J.J., Lowe D.G. (2008). Informed visual search: Combining attention and object recognition. Proceedings of the 2008 IEEE International Conference on Robotics and Automatio.

[45] Roy S.D., Chaudhury S., Banerjee S. (2004). Active recognition through next view planning: A survey. Pattern Recognit..

[46] Atanasov N., Le Ny J., Daniilidis K., Pappas G.J. (2015). Decentralized active information acquisition: Theory and application to multi-robot SLAM. Proceedings of the 2015 IEEE International Conference on Robotics and Automation (ICRA).

[47] Connolly C. (1985). The Determination of Next Best Views. Proceedings of the 1985 IEEE International Conference on Robotics and Automation.

[48] Pito R. (1999). A solution to the next best view problem for automated surface acquisition. IEEE Trans. Pattern Anal. Mach. Intell..

[49] Mendoza M., Vasquez-Gomez J.I., Taud H., Sucar L.E., Reta C. (2020). Supervised learning of the next-best-view for 3d object reconstruction. Pattern Recognit. Lett..

[50] Pan S., Hu H., Wei H., Dengler N., Zaenker T., Dawood M., Bennewitz M. (2025). Integrating One-Shot View Planning With a Single Next-Best View via Long-Tail Multiview Sampling. IEEE Trans. Robot..

[51] Hu B., Chang C., Ge J., Namgung M., Lin X., Krieger A., Mohsenin T. (2026). OA-NBV: Occlusion-Aware Next-Best-View Planning for Human-Centered Active Perception on Mobile Robots. arXiv.

[52] Yang J., Waslander S.L. (2022). Next-best-view prediction for active stereo cameras and highly reflective objects. Proceedings of the 2022 International Conference on Robotics and Automation (ICRA).

[53] Burusa A.K., van Henten E.J., Kootstra G. (2024). Gradient-based local next-best-view planning for improved perception of targeted plant nodes. Proceedings of the 2024 IEEE International Conference on Robotics and Automation (ICRA).

[54] Yu X., Chen C.W. Semantic-aware next-best-view for multi-DoFs mobile system in search-and-acquisition based visual perception. Proceedings of the 32nd ACM International Conference on Multimedia.

[55] Hutchinson S., Hager G.D., Corke P.I. (1996). A tutorial on visual servo control. IEEE Trans. Robot. Autom..

[56] Vogel J., de Freitas N. (2008). Target-directed attention: Sequential decision-making for gaze planning. Proceedings of the 2008 IEEE International Conference on Robotics and Automation.

[57] Papanikolopoulos N.P., Khosla P.K., Kanade T. (1993). Visual tracking of a moving target by a camera mounted on a robot: A combination of control and vision. IEEE Trans. Robot. Autom..

[58] Frintrop S., Jensfelt P. (2008). Attentional landmarks and active gaze control for visual SLAM. IEEE Trans. Robot..

[59] Velez J., Hemann G., Huang A., Posner I., Roy N. (2011). Planning to perceive: Exploiting mobility for robust object detection. Proc. Int. Conf. Autom. Plan. Sched..

[60] Bartolomei L., Teixeira L., Chli M. (2021). Semantic-aware active perception for uavs using deep reinforcement learning. Proceedings of the 2021 IEEE/RSJ International Conference on Intelligent Robots and Systems (IROS).

[61] Wang Z., Chen H., Zhang S., Lou Y. (2024). Active view planning for visual slam in outdoor environments based on continuous information modeling. IEEE/ASME Trans. Mechatron..

[62] Nelson B.J., Khosla P.K. (1997). Visually guided manipulation using active camera-lens systems. Proceedings of the International Conference on Robotics and Automation.

[63] Jiang H., Huang B., Wu R., Li Z., Garg S., Nayyeri H., Wang S., Li Y. (2024). Roboexp: Action-conditioned scene graph via interactive exploration for robotic manipulation. arXiv.

[64] Yan Z., Su F., Wang M., Hu R., Zhang H., Huang H. (2023). Interaction-Driven Active 3D Reconstruction with Object Interiors. ACM Trans. Graph..

[65] Dutta A., Burdet E., Kaboli M. (2025). Predictive Visuo-Tactile Interactive Perception Framework for Object Properties Inference. IEEE Trans. Robot..

[66] He Y., Zhang R., Shen T., Liu C., Nie Q. (2026). Towards Exploratory and Focused Manipulation with Bimanual Active Perception: A New Problem, Benchmark and Strategy. arXiv.

[67] Zhou B., Xu H., Shen S. (2023). Racer: Rapid collaborative exploration with a decentralized multi-uav system. IEEE Trans. Robot..

[68] Chiun J., Zhang S., Wang Y., Cao Y., Sartoretti G. (2025). MARVEL: Multi-Agent Reinforcement Learning for constrained field-of-View multi-robot Exploration in Large-scale environments. Proceedings of the 2025 IEEE International Conference on Robotics and Automation (ICRA).

[69] Zhang X., Wang D., Han S., Li W., Zhao B., Wang Z., Duan X., Fang C., Li X., He J. (2023). Affordance-driven next-best-view planning for robotic grasping. arXiv.

[70] Wang S., Li Y., Guo C., Tan G. (2026). Learning hierarchical uncertainty from hybrid representations for neural active reconstruction. Pattern Recognit..

[71] Carrillo H., Reid I., Castellanos J.A. (2012). On the comparison of uncertainty criteria for active SLAM. Proceedings of the 2012 IEEE International Conference on Robotics and Automation.

[72] Rodríguez-Arévalo M.L., Neira J., Castellanos J.A. (2018). On the importance of uncertainty representation in active SLAM. IEEE Trans. Robot..

[73] Burusa A.K., van Henten E.J., Kootstra G. (2024). Attention-driven next-best-view planning for efficient reconstruction of plants and targeted plant parts. Biosyst. Eng..

[74] Burusa A.K., Scholten J., Wang X., Rapado-Rincon D., van Henten E.J., Kootstra G. (2024). Semantics-aware next-best-view planning for efficient search and detection of task-relevant plant parts. Biosyst. Eng..

[75] Dai N., Fang J., Yuan J., Liu X. (2026). LARIS: A local-state active reasoning and integrated switching framework for robotic harvesting under partial observability. Comput. Electron. Agric..

[76] Wang G., Li H., Zhang S., Guo D., Liu Y., Liu H. (2025). Observe Then Act: Asynchronous Active Vision-Action Model for Robotic Manipulation. IEEE Robot. Autom. Lett..

[77] Dai Y., Chen S., Yang K., Hu D., Xie P., Li G., Shen Y., Wang G. (2025). Active-Perceptive Language-Oriented Grasp Policy for Heavily Cluttered Scenes. IEEE Robot. Autom. Lett..

[78] Lan Q., Li Z., Zhang H., Yang J. (2026). Optical active perception with 3D Gaussian splatting enables autonomous instrument insertion in robotic vitreoretinal surgery. Opt. Lett..

[79] Kennis G., Arai S. (2026). HEAPGrasp: Hand-Eye Active Perception to Grasp Objects With Diverse Optical Properties. IEEE Robot. Autom. Lett..

[80] Qin L., Wang M., Li P., Zhou W., Li H. (2025). Active perception meets rule-guided rl: A two-phase approach for precise object navigation in complex environments. Proceedings of the 2025 IEEE/CVF International Conference on Computer Vision (ICCV).

[81] Wang Y., Tian G., Shao X. (2026). Approaching for manipulation: Robot termination pose generation via active object detection and task-oriented logical reasoning. Expert Syst. Appl..

[82] Guo Y., Sun J., Zhang R., Jiang Z., Mi Z., Yao C., Ban X., Obaidat M.S. (2024). An Object-Driven Navigation Strategy Based on Active Perception and Semantic Association. IEEE Robot. Autom. Lett..

[83] Wang Y., Tian G. (2026). Object search strategy for service robots with knowledge-based viewpoint selection and hierarchical action decisions. Expert Syst. Appl..

[84] Strand S.H., Wiedemann T., Burczek B., Shutin D. (2026). Enhancing UAV Search Under Occlusion Using Next Best View Planning. IEEE J. Sel. Top. Appl. Earth Obs. Remote Sens..

[85] Sethi N., Lodel M., Ferranti L., Babuska R., Alonso-Mora J. (2026). STEM: Semantic target search and exploration using MAVs in cluttered environments. Auton. Robot..

[86] Hu Y., Peng J., Liu S., Ge J., Liu S., Chen S. (2024). Communication-efficient collaborative perception via information filling with codebook. Proceedings of the 2024 IEEE/CVF Conference on Computer Vision and Pattern Recognition (CVPR).

[87] Huang J., Zhu J., Li R., Zhao Z., Zhang H. (2025). Select2Drive: Pragmatic Communications for Real-Time Collaborative Autonomous Driving. IEEE Trans. Intell. Transp. Syst..

[88] Zhou S., Jia Y., Mao R., Nan Z., Sun Y., Niu Z. (2024). Task-Oriented Wireless Communications for Collaborative Perception in Intelligent Unmanned Systems. IEEE Netw..

[89] Zhang C., Steinhauser F., Hinz G., Knoll A. (2024). Occlusion-Aware Planning for Autonomous Driving With Vehicle-to-Everything Communication. IEEE Trans. Intell. Veh..

[90] Huang X., Ying Y., Dong W. (2024). CEASE: Collision-Evaluation-Based Active Sense System for Collaborative Robotic Arms. IEEE Trans. Instrum. Meas..

[91] Liu Y., Jia T., Zhang H., Yang G., Wang H., Chen D. (2025). RASP: Robot Active Scene Perception With Joint Viewpoint Planning and Depth Completion in Cluttered Environments. IEEE Trans. Autom. Sci. Eng..

[92] Cho D., Jang Y., Xu D., Ha S. (2026). EgoAVFlow: Robot Policy Learning With Active Vision From Human Egocentric Videos via 3D Flow. IEEE Robot. Autom. Lett..

[93] Wang G., Liu Y., Liu H. (2025). One model, two skills: Active vision and action learning model for robotic manipulation. Sci.-China-Inf. Sci..

[94] Border R., Gammell J.D. (2024). The surface edge explorer (SEE): A measurement-direct approach to next best view planning. Int. J. Robot. Res..

[95] Carlone L., Du J., Kaouk Ng M., Bona B., Indri M. (2014). Active SLAM and exploration with particle filters using Kullback-Leibler divergence. J. Intell. Robot. Syst..

[96] Yang Z., Sang A.W.Y., Muthugala M.A.V.J., Elara M.R. (2026). Mutual information-based hierarchical NBV decision for active semantic visual SLAM under dynamic environments. Sci. Rep..

[97] Jiang W., Lei B., Daniilidis K. (2024). Fisherrf: Active view selection and mapping with radiance fields using fisher information. Proceedings of the European Conference on Computer Vision.

[98] Lauri M., Pajarinen J., Peters J., Frintrop S. (2020). Multi-sensor next-best-view planning as matroid-constrained submodular maximization. IEEE Robot. Autom. Lett..

[99] Bai R., Yuan S., Guo H., Yin P., Yau W.Y., Xie L. (2024). Multi-robot active graph exploration with reduced pose-slam uncertainty via submodular optimization. Proceedings of the 2024 IEEE/RSJ International Conference on Intelligent Robots and Systems (IROS).

[100] Yan Z., Yang H., Zha H. (2023). Active neural mapping. Proceedings of the 2023 IEEE/CVF International Conference on Computer Vision (ICCV).

[101] Popović M., Ott J., Rückin J., Kochenderfer M.J. (2024). Learning-based methods for adaptive informative path planning. Robot. Auton. Syst..

[102] Bircher A., Kamel M., Alexis K., Oleynikova H., Siegwart R. (2016). Receding horizon “next-best-view” planner for 3d exploration. Proceedings of the 2016 IEEE International Conference on Robotics and Automation (ICRA).

[103] Atanasov N., Sankaran B., Le Ny J., Pappas G.J., Daniilidis K. (2014). Nonmyopic view planning for active object classification and pose estimation. IEEE Trans. Robot..

[104] Chen Z., Zhao J., Yang M., Zhou W., Li H. (2024). Optimizing camera motion with MCTS and target motion modeling in multi-target active object tracking. ACM Trans. Multimed. Comput. Commun. Appl..

[105] Avni O., Borrelli F., Katzir G., Rivlin E., Rotstein H. (2008). Scanning and tracking with independent cameras—a biologically motivated approach based on model predictive control. Auton. Robot..

[106] Chen Y., Huang S., Fitch R. (2020). Active SLAM for mobile robots with area coverage and obstacle avoidance. IEEE/ASME Trans. Mechatron..

[107] Allibert G., Courtial E., Chaumette F. (2010). Predictive control for constrained image-based visual servoing. IEEE Trans. Robot..

[108] Kerr J., Hari K., Weber E., Kim C.M., Yi B., Bonnen T., Goldberg K., Kanazawa A. (2025). Eye, robot: Learning to look to act with a bc-rl perception-action loop. arXiv.

[109] Naveed K., Hussain W., Hussain I., Lee D., Anjum M.L. (2025). Help Me Through: Imitation Learning Based Active View Planning to Avoid SLAM Tracking Failures. IEEE Trans. Robot..

[110] Cheng R., Agarwal A., Fragkiadaki K. (2018). Reinforcement learning of active vision for manipulating objects under occlusions. Proceedings of the Conference on Robot Learning.

[111] Placed J.A., Castellanos J.A. (2020). A deep reinforcement learning approach for active SLAM. Appl. Sci..

[112] Li J., Xu J., Zhong F., Kong X., Qiao Y., Wang Y. (2020). Pose-assisted multi-camera collaboration for active object tracking. Proc. AAAI Conf. Artif. Intell..

[113] Huang B., Yu J., Jain S. (2023). EARL: Eye-on-hand reinforcement learner for dynamic grasping with active pose estimation. Proceedings of the 2023 IEEE/RSJ International Conference on Intelligent Robots and Systems (IROS).

[114] Hu E., Wang J., Yuan X., Luo F., Li M., Lambrechts G., Rybkin O., Jayaraman D. (2026). Real-World Reinforcement Learning of Active Perception Behaviors. Adv. Neural Inf. Process. Syst..

[115] Wang T., Huang Q., Liu A., Qin G., Zhang J., Yang Y., Cheng Y. (2026). Dual-resolution next-best-view planning for manipulator-based autonomous mapping in fusion reactor maintenance. Fusion Eng. Des..

[116] Kim A., Eustice R.M. Next-best-view visual SLAM for bounded-error area coverage. Proceedings of the IROS Workshop on Active Semantic Perception.

[117] Kitanov A., Indelman V. (2024). Topological belief space planning for active SLAM with pairwise Gaussian potentials and performance guarantees. Int. J. Robot. Res..

[118] Ammirato P., Poirson P., Park E., Košecká J., Berg A.C. (2017). A dataset for developing and benchmarking active vision. Proceedings of the 2017 IEEE International Conference on Robotics and Automation (ICRA).

[119] Savva M., Kadian A., Maksymets O., Zhao Y., Wijmans E., Jain B., Straub J., Liu J., Koltun V., Malik J. (2019). Habitat: A platform for embodied ai research. Proceedings of the 2019 IEEE/CVF International Conference on Computer Vision (ICCV).

[120] Deitke M., Han W., Herrasti A., Kembhavi A., Kolve E., Mottaghi R., Salvador J., Schwenk D., VanderBilt E., Wallingford M. (2020). Robothor: An open simulation-to-real embodied ai platform. Proceedings of the 2020 IEEE/CVF Conference on Computer Vision and Pattern Recognition (CVPR).

[121] Dhami H., Sharma V.D., Tokekar P. (2023). Pred-nbv: Prediction-guided next-best-view planning for 3d object reconstruction. Proceedings of the 2023 IEEE/RSJ International Conference on Intelligent Robots and Systems (IROS).

[122] Cai Y., Medioni G. (2016). Persistent people tracking and face capture using a PTZ camera. Mach. Vis. Appl..

[123] Schaub H., Wolff C., Hoh M., Schoettl A. (2024). Probabilistic Closed-Loop Active Grasping. IEEE Robot. Autom. Lett..

[124] An B., Geng Y., Chen K., Li X., Dou Q., Dong H. (2024). Rgbmanip: Monocular image-based robotic manipulation through active object pose estimation. Proceedings of the 2024 IEEE International Conference on Robotics and Automation (ICRA).

[125] Chuang I., Lee A., Gao D., Naddaf-Sh M.M., Soltani I. (2025). Active vision might be all you need: Exploring active vision in bimanual robotic manipulation. Proceedings of the 2025 IEEE International Conference on Robotics and Automation (ICRA).

[126] Lehnert C., Tsai D., Eriksson A., McCool C. (2019). 3d move to see: Multi-perspective visual servoing towards the next best view within unstructured and occluded environments. Proceedings of the 2019 IEEE/RSJ International Conference on Intelligent Robots and Systems (IROS).

[127] Koc C., Sariel S. (2024). Object-aware interactive perception for tabletop scene exploration. Robot. Auton. Syst..

[128] Li P., Huang Y., Chang W., Zhou C., Wang S., Yu J., Wu Z. (2025). Active SLAM With Dynamic Viewpoint Optimization for Robust Visual Navigation. IEEE Trans. Instrum. Meas..

[129] Mo Y., Vijay R., Rufus R., de Boer N., Kim J., Yu M. (2024). Enhanced Perception for Autonomous Vehicles at Obstructed Intersections: An Implementation of Vehicle to Infrastructure (V2I) Collaboration. Sensors.

[130] Zhang W., Gu Y., Zhao B., Deng Q., Chen X., Shi Y., Jiang L., Cao S., Jiang Z., Mao R. (2025). Near-Sensor LiDAR and Visual Feature Extraction and Communication for Low-Latency Roadside Cooperative Perception. IEEE Internet Things J..

[131] Dao M.Q., Berrio J.S., Fremont V., Shan M., Hery E., Worrall S. (2024). Practical Collaborative Perception: A Framework for Asynchronous and Multi-Agent 3D Object Detection. IEEE Trans. Intell. Transp. Syst..

[132] Ying Y., Huang X., Dong W. (2024). Multi-view active sensing for human-robot interaction via hierarchically connected tree. Sens. Actuators-A-Phys..

[133] Halverson T., Hornof A.J. (2011). A computational model of “active vision” for visual search in human–computer interaction. Hum.-Interact..

[134] Reale M.J., Canavan S., Yin L., Hu K., Hung T. (2011). A multi-gesture interaction system using a 3-D iris disk model for gaze estimation and an active appearance model for 3-D hand pointing. IEEE Trans. Multimed..

[135] Das D., Rashed M.G., Kobayashi Y., Kuno Y. (2015). Supporting human–robot interaction based on the level of visual focus of attention. IEEE Trans. Hum.-Mach. Syst..

[136] Ognibene D., Foulsham T., Marchegiani L., Farinella G.M. (2022). Active vision and perception in human-robot collaboration. Front. Neurorobotics.

[137] Fan J., Zheng P., Li S. (2022). Vision-based holistic scene understanding towards proactive human–robot collaboration. Robot. Comput.-Integr. Manuf..

[138] Wang P., Ma R., Yang Z., Hao Q. (2024). Robotic Camera Array Motion Planning for Multiple Human Face Tracking Based on Reinforcement Learning. IEEE Sens. J..

[139] Foresti G.L., Micheloni C., Snidaro L., Remagnino P., Ellis T. (2005). Active video-based surveillance system: The low-level image and video processing techniques needed for implementation. IEEE Signal Process. Mag..

[140] Yang X., Zeng D., Wang X., Ye H., Xie X., Zhao Q., Zhu J., Li S. (2026). Toward Real-Time UAV Tracking With Adaptive and Background-Aware Vision Transformers. IEEE Trans. Circuits Syst. Video Technol..

[141] Mo J., He F., Zhong P., Zhao Q., Zeng D., Huang L., Li S., Shen X. (2026). UniTrack: Unifying day and night tracking with continual learning. Inf. Sci..

[142] Lee H., Lim D., Han S.H., Lee J., Lee S.h., Park J. (2025). An empirical study of PTZ camera-based drone tracking under latency and detector challenges. Mach. Vis. Appl..

[143] Roshandel N., Scholz C., Cao H.L., Cao H.G., Amighi M., Firouzipouyaei H., Burkiewicz A., Menet S., Ballen-Moreno F., Sisavath D.W. (2025). mmPrivPose3D: A RaDAR-Based Approach to Privacy-Compliant Pose Estimation and Gesture Command Recognition in Human-Robot Collaboration. IEEE Sens. J..

[144] Agraz C.N., Hinrichs P., Eichelberg M., Hein A. (2025). Is the Robot Spying on me? A Study on Perceived Privacy in Telepresence Scenarios in a Care Setting with Mobile and Humanoid Robots. Int. J. Soc. Robot..

[145] Ciccone F., Ceruti A. (2025). Real-Time Search and Rescue with Drones: A Deep Learning Approach for Small-Object Detection Based on YOLO. Drones.

[146] Boiteau S., Vanegas F., Gonzalez F. (2024). Framework for Autonomous UAV Navigation and Target Detection in Global-Navigation-Satellite-System-Denied and Visually Degraded Environments. Remote Sens..

[147] Tallamraju R., Price E., Ludwig R., Karlapalem K., Bülthoff H.H., Black M.J., Ahmad A. (2019). Active perception based formation control for multiple aerial vehicles. IEEE Robot. Autom. Lett..

[148] Zhou Y., Xiao J., Zhou Y., Loianno G. (2022). Multi-robot collaborative perception with graph neural networks. IEEE Robot. Autom. Lett..

[149] Huang C.H., Chen Y.C., Hsu C.Y., Yang J.Y., Chang C.H. (2024). FPGA-based UAV and UGV for search and rescue applications: A case study. Comput. Electr. Eng..

[150] Zhao J., Li Q., Chi P., Wang Y. (2025). Active Vision-Based UAV Swarm With Limited FOV Flocking in Communication-Denied Scenarios. IEEE Trans. Instrum. Meas..

[151] Thummalapeta M., Chen S.W., Liu Y.C. (2024). Importance-based distributed persistent coverage strategy over environmental hotspots using quadrotors: Algorithm and experiments. J. Frankl.-Inst.-Eng. Appl. Math..

[152] Wang X., Burusa A.K., Rapado-Rincón D., Versmissen T.A., van Henten E.J., Kootstra G. (2025). Multiple object tracking with multi-view active vision to effectively find plant nodes in a cluttered tomato greenhouse. Comput. Electron. Agric..

[153] Faghir Hagh S., Amngostar P., Burns D., Figueiredo R., Ku Y.J., Cianci-Gaskill J., McMurray S.E., Huston D., Xia T. (2026). Internet of Drones System for Real-Time Wireless Water Quality Sensing, 2-D Mapping, 3-D Depth Profiling, and Intelligent Sampling. IEEE Internet Things J..

[154] Bucci A., Ridolfi A. (2026). Multi-session perception-aware coverage path planning for active semantic SLAM and automatic change detection. Ocean. Eng..

[155] Arora A., Furlong P.M., Fitch R., Sukkarieh S., Fong T. (2019). Multi-modal active perception for information gathering in science missions. Auton. Robot..

[156] Tran V.P., Perera A.G., Garratt M.A., Kasmarik K., Anavatti S.G. (2025). Active Sensing Strategy: Multi-Modal, Multi-Robot Source Localization and Mapping in Real-World Settings With Fixed One-Way Switching. J. Field Robot..

[157] Mercado M.A., Kasuga H., Motegi Y., Maki T. (2026). Reactive Vision-Based Benthic Monitoring Method for an Underactuated AUV. IEEE J. Ocean. Eng..

[158] Nowakowski M., Kurylo J., Dang P.H. (2023). Camera based AI models used with LiDAR data for improvement of detected object parameters. Proceedings of the International Conference on Modelling and Simulation for Autonomous Systems.

[159] Yang C., Chen Y., Tian H., Tao C., Zhu X., Zhang Z., Huang G., Li H., Qiao Y., Lu L. (2023). Bevformer v2: Adapting modern image backbones to bird’s-eye-view recognition via perspective supervision. Proceedings of the 2023 IEEE/CVF Conference on Computer Vision and Pattern Recognition (CVPR).

